# A hybrid ViT-L/32–MaxViT-L architecture with adaptive gated fusion for multiclass gastrointestinal disease detection and multi-method *post-hoc* explainability

**DOI:** 10.3389/fendo.2026.1869015

**Published:** 2026-07-14

**Authors:** Shahid Mohammad Ganie, Pijush Kanti Dutta Pramanik, Zhongming Zhao

**Affiliations:** 1 Department of Health Information Management and Technology, College of Applied Medical Sciences, King Faisal University, Al-Ahsa, Saudi Arabia; 2 School of Computer Applications and Technology Galgotias University Greater Noida, Uttar Pradesh, India; 3 McWilliams School of Biomedical Informatics, The University of Texas Health Science Center at Houston, Houston, TX, United States

**Keywords:** adaptive gated fusion, endoscopic image classification, explainable AI, gastrointestinal disease detection, hybrid transformer architecture, vision transformer

## Abstract

**Introduction:**

Accurate detection of gastrointestinal diseases from endoscopic images remains challenging due to substantial inter-class similarity, intra-class variability, and heterogeneous lesion presentation. While convolutional neural networks (CNNs) effectively capture fine mucosal textures, their limited receptive field restricts global contextual reasoning. Conversely, transformer architectures model long-range dependencies but may underrepresent localized structural detail.

**Methods:**

This study proposes a dual-branch hybrid transformer framework that integrates global token-level reasoning from ViT-L/32 with hierarchical spatial modeling from MaxViT-L. An adaptive gated fusion mechanism is introduced to dynamically regulate the relative contribution of local and global representations on a per-sample basis, enabling content-aware cross-scale integration. The model was evaluated on a multi-class gastrointestinal endoscopic dataset using comprehensive quantitative metrics, class-wise analysis, ablation studies, confidence interval estimation, and statistical validation. Post-hoc explainability was further investigated using multiple complementary XAI techniques.

**Results:**

The proposed framework demonstrated consistently high accuracy, sensitivity, specificity, and area under the ROC curve across eight gastrointestinal disease categories, outperforming several state-of-the-art transformer and hybrid architectures. The XAI analyses further confirmed lesion-focused activation consistency and stable decision-making.

**Discussion:**

The findings indicate that adaptive local–global feature integration through gated fusion enhances both diagnostic performance and interpretability, supporting the potential of hybrid transformer architectures for reliable computer-aided gastrointestinal disease classification.

## 1 Introduction

Gastrointestinal diseases represent a significant global health burden, including inflammatory disorders, precancerous lesions, and malignant conditions that require timely and accurate detection for effective clinical management. Endoscopic imaging remains the primary diagnostic modality for identifying mucosal abnormalities within the esophagus, stomach, and colon. However, interpretation of endoscopic findings is inherently operator-dependent and subject to inter-observer variability, particularly when distinguishing subtle inflammatory changes, small polyps, dyed margins, or visually similar anatomical structures. Missed or misclassified lesions can delay treatment and adversely affect patient outcomes. Consequently, computer-aided diagnostic systems have emerged as a promising strategy to enhance diagnostic consistency, sensitivity, and workflow efficiency in gastrointestinal screening and clinical practice.

Deep learning has significantly advanced automated gastrointestinal image analysis over the past decade. Convolutional Neural Networks (CNNs) have demonstrated strong performance in polyp detection, ulcer recognition, bleeding identification, and multi-class endoscopic disease classification due to their capacity for hierarchical feature extraction (1, 2). CNN-based approaches effectively capture local texture patterns and structural irregularities, which are critical in identifying protrusions, mucosal disruptions, and inflammatory regions (3). Nonetheless, CNNs inherently rely on localized receptive fields, which can limit their ability to model global contextual dependencies (4). In endoscopic imaging, diagnostic interpretation often depends not only on localized lesions but also on surrounding mucosal context and anatomical continuity, where long-range spatial relationships may influence classification (5, 6).

Recent exploration of advanced deep learning architectures has introduced transformer-based models into gastrointestinal disease analysis (7). Vision Transformers (ViTs), adapted from natural language processing, leverage self-attention mechanisms to model global contextual relationships across image patches. By treating image patches as tokens and computing attention across the full sequence, ViTs enable holistic reasoning that extends beyond convolutional locality. Emerging applications in medical imaging demonstrate the growing relevance of attention-based modeling. In gastrointestinal imaging specifically, transformer variants have been explored for polyp detection and endoscopic classification, indicating potential advantages in capturing complex mucosal patterns and anatomical variability.

Despite these advancements, important limitations persist. At a general level, many transformer architectures emphasize either global token-level reasoning or hierarchical spatial modeling but rarely integrate both in a structured and adaptive manner (8, 9). Pure ViT models may underrepresent fine-grained local features, while hierarchical transformers may not fully exploit long-range contextual dependencies (10). Moreover, the growing complexity of deep architectures has amplified concerns regarding interpretability, as most models operate as opaque decision systems with limited transparency (11, 12).

These limitations are particularly relevant in gastrointestinal disease detection. Gastrointestinal pathologies often present as a combination of localized structural abnormalities and diffuse inflammatory patterns (13, 14). For example, ulcerative colitis involves widespread mucosal changes, whereas polyps and dyed lesions are spatially localized yet context-sensitive. Effective classification therefore requires simultaneous modeling of fine-grained spatial cues and global anatomical context. Furthermore, in endoscopic applications where diagnostic decisions directly influence biopsy, resection, or surveillance strategies, the lack of interpretable reasoning can hinder clinical trust and adoption. Although explainable AI (XAI) methods are commonly applied *post-hoc*, they are often treated as auxiliary visualization tools rather than systematically evaluated components aligned with model behavior.

To address these challenges, this study proposes a Hybrid ViT-L/32 + MaxViT-L architecture with adaptive gated fusion for multiclass gastrointestinal disease detection. The central hypothesis is that global token-level contextual reasoning and hierarchical multi-axis spatial attention provide complementary diagnostic cues, and that their dynamic integration through a learnable gating mechanism can enhance class separability, stabilize generalization, and maintain clinically coherent interpretability.

**Figure 1:**
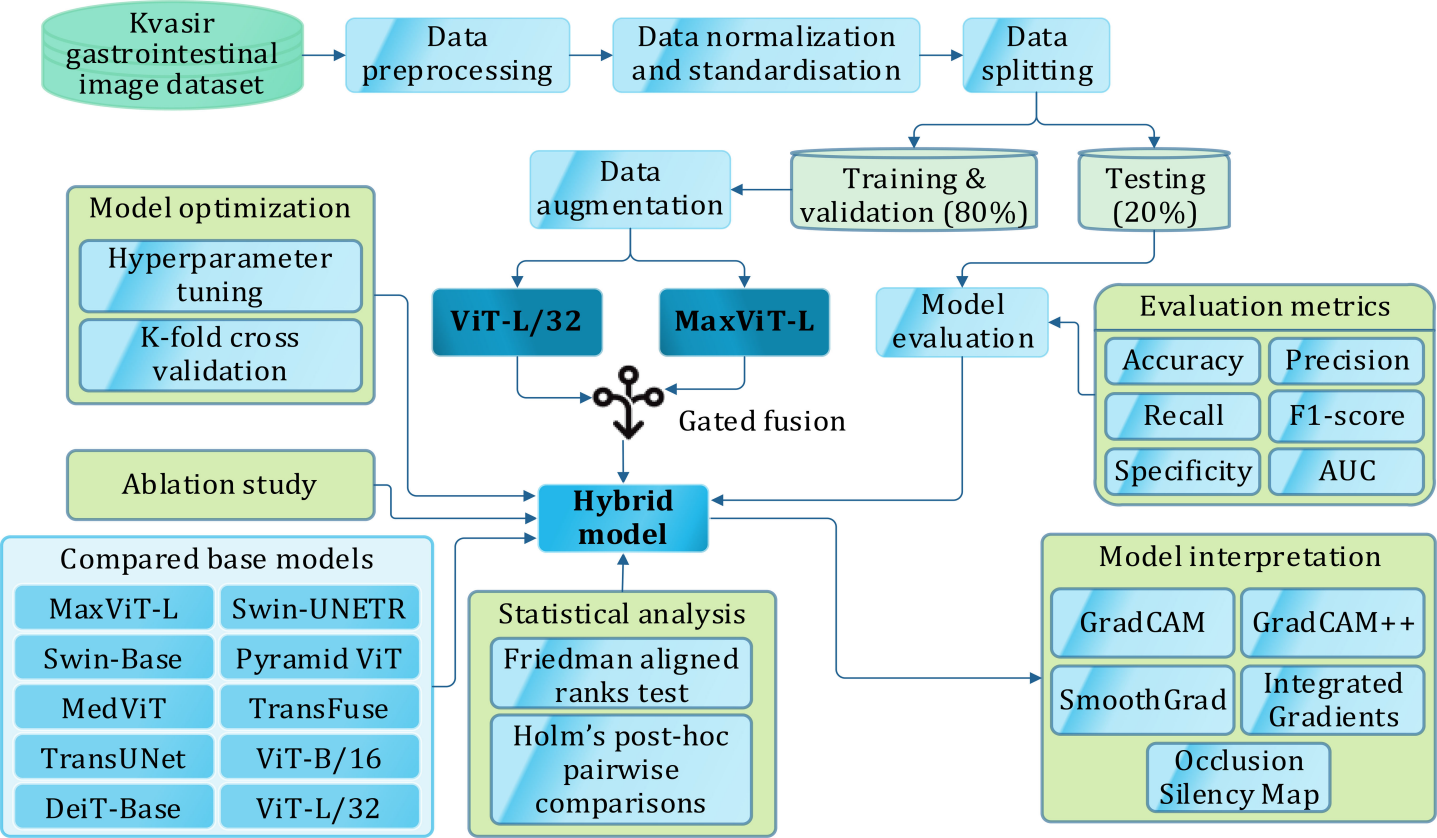
Overview of the proposed framework.

The objectives of this work are threefold: (i) to design a dual-stream transformer architecture integrating ViT-L/32 and MaxViT-L to capture both long-range contextual dependencies and fine-grained mucosal structures; (ii) to develop an adaptive gated fusion mechanism that dynamically balances global and local feature representations on a per-sample basis; and (iii) to achieve robust multiclass discrimination across eight clinically relevant gastrointestinal conditions while ensuring stable optimization and reliable interpretability. The overall workflow of the proposed framework, including data preparation, hybrid model development, evaluation, statistical validation, and explainability analysis, is illustrated in **Figure 1**.

The primary contributions of this study are summarized as follows:

1. Hybrid transformer architecture: We introduce a dual-stream framework combining global token-based ViT modeling with hierarchical multi-axis attention, enabling comprehensive representation learning for endoscopic images.
2. Adaptive gated feature fusion: A sample-dependent gated fusion mechanism is proposed to dynamically weight complementary features, reducing redundancy and enhancing discriminative capacity.
3. Robust multiclass performance: The model achieves strong performance across accuracy, precision, recall, F1-score, specificity, and AUC for eight gastrointestinal categories within a unified framework.
4. Improved class-wise reliability: Detailed confusion matrix and ROC analyses demonstrate reliable differentiation between visually similar disease categories and normal anatomical structures.
5. Stable optimization of large transformers: Leveraging pretrained backbones and controlled fine-tuning strategies enables stable convergence with minimal overfitting on moderate-scale medical datasets.
6. Comprehensive comparative validation: Extensive benchmarking against state-of-the-art transformer and hybrid architectures establishes a strong transformer-based baseline for gastrointestinal disease detection.
7. Statistical significance analysis: A rigorous statistical evaluation framework is incorporated using Friedman’s aligned ranks test with Holm-adjusted *post-hoc* comparisons to validate the significance of performance improvements over competing models.
8. Clinically relevant interpretability: Multi-method *post-hoc* explainability analysis demonstrates lesion-focused activation patterns, enhancing transparency and supporting potential deployment in decision-support systems.

The remainder of this paper is organized as follows. Section 2 reviews the relevant literature on deep learning in gastrointestinal imaging, transformer architectures, hybrid local–global fusion, and explainable AI. Section 3 describes the dataset and data preparation procedures, including preprocessing and augmentation. Section 4 presents the proposed hybrid architecture, detailing the ViT and MaxViT branches, shared latent projection, adaptive gated fusion, and classifier design. Section 5 formulates the mathematical model and training objective. Section 6 reports the experimental results, including convergence behavior, quantitative metrics, class-wise analysis, ROC evaluation, and comparisons with transformer-based and state-of-the-art models. Section 7 provides statistical validation, and Section 8 presents the ablation study. Section 9 examines interpretability using multi-method XAI analysis. Section 10 discusses clinical implications, Section 11 outlines limitations and future directions, and Section 12 concludes the study.

## 2 Related work

The rapid evolution of deep learning in gastrointestinal imaging spans convolutional models, transformer architectures, hybrid local–global fusion strategies, and explainability frameworks. This section critically examines prior work across these dimensions to position the methodological and interpretive contributions of the proposed gated hybrid transformer model.

### 2.1 Deep learning for gastrointestinal endoscopic analysis

Deep learning, particularly CNNs, now forms the backbone of most computer-aided diagnostic systems in gastrointestinal endoscopy. Over the past decade, a wide range of deep learning and image-processing frameworks have been proposed to automate detection, classification, and characterization of gastrointestinal tract disorders, with many systems approaching or exceeding expert-level performance under controlled evaluation settings (15). Research efforts span real-time lesion detection, multi-class disease classification, wireless capsule endoscopy analysis, and artifact-aware modeling, reflecting the diversity and clinical complexity of endoscopic imaging tasks.

In polyp and cancer detection across both upper and lower gastrointestinal tracts, CNN-based object detectors have demonstrated high sensitivity with real-time throughput. Single-shot detector (SSD)-based systems and commercially deployed platforms such as GI Genius report sensitivities exceeding 90% while maintaining processing speeds of up to 50 frames per second (16–18). Beyond binary detection, more advanced frameworks have attempted lesion subtyping and staging. For example, a large-scale gastric neoplasm system capable of classifying six lesion categories and estimating T stage achieved approximately 95% per-patient detection accuracy and around 85% T-stage classification accuracy, illustrating the progression from simple detection to clinically actionable stratification (19).

Wireless capsule endoscopy (WCE) represents another major application domain, where image heterogeneity and limited field control pose additional challenges. Custom CNN architectures such as GINet have reported approximately 99% classification accuracy for bleeding, ulcers, angiectasia, and lymphangiectasia, with Grad-CAM employed to localize salient regions within capsule frames (20). A meta-analysis of 16 WCE studies further reported pooled sensitivity and specificity values in the range of 0.96–1.00 for ulcer, bleeding, and polyp/cancer detection, supporting the clinical feasibility of DL-assisted capsule analysis (21).

For multi-class disease classification from still endoscopic images, transfer learning using established CNN backbones—including ResNet, EfficientNet, Xception, Inception-ResNet, and DenseNet—has become standard practice. Studies on datasets such as Kvasir and HyperKvasir routinely report classification accuracies between 95% and 99% across multi-class gastrointestinal categories (22–24). Ensemble and fusion-based CNN models further improve performance by combining complementary feature extractors. Multi-feature CNN architectures and deep ensembles have reported higher accuracies approaching and AUC, indicating strong separability across disease classes (25, 26).

### 2.2 Transformer architectures in medical image classification

Transformer architectures have significantly influenced medical image classification by enabling explicit modeling of long-range contextual dependencies through self-attention mechanisms (27, 28). Their adoption reflects an architectural contrast with CNNs: while CNNs are inherently effective at capturing localized texture and edge-based cues, transformers operate on tokenized representations that allow direct interaction between spatially distant regions. In medical imaging—where anatomical structures span multiple scales and pathological signatures may be spatially distributed—both localized detail and global structural context are often required for reliable discrimination (29–31).

The ViT introduced a patch-based representation framework in which images are decomposed into fixed-size tokens and processed through global self-attention layers (32). Applications in tumor classification and COVID-19 diagnosis demonstrate that ViT-based models can better capture distributed pathological patterns and inter-regional dependencies compared with purely convolutional approaches (33, 34). Nonetheless, standard ViTs incur quadratic computational complexity with respect to token number and lack the spatial inductive biases embedded in convolutional filters, which can hinder performance on relatively small or moderately sized medical datasets (35, 36).

To mitigate these limitations, hierarchical transformer variants such as the Swin Transformer (Swin-T) restrict attention to local windows while permitting cross-window interaction via shifted mechanisms (36, 37). This design reduces computational overhead and supports multi-scale feature abstraction, which is particularly relevant in retinal imaging, musculoskeletal analysis, and pancreatic tumor classification (36–38). However, window-constrained attention may limit unrestricted global reasoning, and fine-grained discrimination in subtle diagnostic tasks remains challenging under purely hierarchical formulations (31, 39).

Hybrid CNN–Transformer architectures attempt to reconcile these modeling properties by combining convolutional locality with global self-attention (29, 30). Sequential configurations apply CNN-based feature extraction followed by transformer-based contextual refinement (35, 40), whereas parallel dual-branch frameworks maintain distinct local and global streams prior to fusion (31, 39, 41). Cross-attention modules further facilitate interaction between representations, enabling more flexible information exchange in heterogeneous medical image settings (42, 43).

Multi-scale representation learning remains central in medical classification tasks, where lesions and structural variations manifest across different spatial resolutions (27, 38). Hierarchical encoders and dual-branch models integrate coarse contextual information with fine structural detail to improve discriminative performance (44, 45). Multi-task strategies that jointly optimize classification and segmentation objectives reinforce both global semantic encoding and boundary-level precision within a unified learning framework (29, 43).

Applications span thyroid nodule analysis, retinal disease detection, histopathological evaluation, breast cancer imaging, and cervical cytology classification, where transformer-based and hybrid approaches report competitive or superior performance compared with conventional CNN baselines (28, 31, 35, 39). Computational trade-offs, data efficiency, and the design of effective feature fusion mechanisms continue to shape architectural development in this domain (30, 35, 36, 44).

### 2.3 Hybrid local–global fusion architectures

The architectural developments discussed above—ranging from global ViT models to hierarchical window-based transformers and CNN–Transformer hybrids—have converged toward a common objective: integrating fine-grained local modeling with global contextual reasoning within a unified framework. Rather than relying exclusively on full self-attention or strictly localized window attention, recent transformer variants incorporate structured fusion mechanisms to balance short-range detail and long-range dependency modeling.

One line of work adopts parallel local–global branches. PLG-ViT processes local window attention and global self-attention in parallel streams and fuses their outputs through convolutional feed-forward modules at each stage (46). MAXFormer applies a similar dual-path design within a U-shaped segmentation architecture, demonstrating the value of explicit local–global separation in medical imaging tasks (47). These approaches preserve complementary feature pathways; however, fusion is largely embedded in architectural design and remains structurally fixed.

A second paradigm integrates local and global reasoning within a single attention operator. The Focal Transformer enables each token to attend densely to nearby regions while summarizing distant regions at a coarser resolution, thereby reducing computational burden while maintaining contextual coverage (48). MaxViT extends this principle through multi-axis attention blocks that combine blocked local attention with dilated global attention in a repeated hierarchical structure (49). Although these designs achieve efficient global–local coverage, the integration occurs implicitly within attention computation rather than through explicit feature weighting or adaptive regulation.

Other architectures employ head-splitting or multi-branch attention strategies, allocating distinct attention heads to global and local feature extraction. GLAFormer separates heads operating on downsampled global features from those attending to local windows, later merging the resulting representations (50). MAFT alternates global pixel-hybrid attention with window-based local attention across multi-attention groups (51). In these configurations, specialization is enforced by design, but the contribution of each pathway is predetermined by architecture rather than dynamically modulated by input characteristics.

Hybrid CNN–Transformer frameworks further emphasize complementary modeling. Channel–spatial transformer blocks integrate convolutional local feature maps with transformer-derived global dependencies (52). GLT-Net and HyperTransXNet combine multiscale CNN feature extraction with transformer-based global spectral modeling and structured fusion strategies (8, 53). Dual-stream architectures such as ConvTransGFusion and NCGLF2 explicitly blend convolutional detail and transformer context using global–local fusion modules (54, 55). In many such systems, fusion is realized through concatenation, additive merging, or stage-wise integration, without explicit regulation of branch dominance on a per-sample basis.

Within medical image classification specifically, hybrid transformer architectures increasingly combine convolutional representations with global self-attention to capture both lesion-level texture and broader anatomical context. ConvTransGFusion processes inputs via ConvNeXt and Swin-T backbones and fuses aligned features through a dual-attention gating module incorporating channel–spatial attention and learnable scaling factors (α, β) (54). HiFuse employs multi-scale parallel local and global branches integrated through hierarchical fusion blocks with spatial and channel attention mechanisms (56), while HEMF extracts multi-scale representations and merges them via mixed-attention and SIRMLP-based modules to reinforce cross-scale consistency (57).

A complementary approach embeds local–global interaction within a single hierarchical backbone. MedViT and MedViTV2 stack convolutional blocks with transformer-based attention modules—including convolutional attention and dilated neighborhood attention—forming structured Local Feature Perception and Global Feature Perception blocks within one network stream (58, 59). Med-Former similarly incorporates an internal Local–Global Transformer module and spatial attention fusion within a unified architecture (60).

Beyond classification accuracy, several works integrate interpretability into hybrid fusion frameworks. EFFResNet-ViT combines CNN-derived local features with ViT-based global tokens and applies Grad-CAM and feature-space visualization to elucidate fused decisions (61). ILAM merges autoencoder-derived fine-grained representations with global tokens and employs modified attention rollout for stable activation mapping (62). An interpretable fully convolutional CNN–Transformer model further produces class-specific evidence maps directly from fused representations, reducing dependence on purely *post-hoc* saliency techniques (63).

### 2.4 Transformer-based models for gastrointestinal disease detection

Building on the broader adoption of transformer and hybrid architectures in medical imaging, transformer-based models have been increasingly applied to gastrointestinal endoscopic analysis. Across tasks including polyp detection, ulcer and inflammatory disease classification, colorectal cancer assessment, WCE analysis, and histopathological evaluation, transformer-driven designs have been investigated to capture both fine mucosal detail and broader anatomical context (15, 64, 65).

Pure transformer formulations have been explored in several gastrointestinal settings. ViT-based models have been applied to colon WCE image classification, where performance superior to DenseNet201 has been reported (95.6% vs. 71.9% accuracy) for distinguishing ulcerative colitis, polyps, esophagitis, and normal colon findings (66). A colorectal cancer detection framework (ViTCol) has incorporated a spatial transformer module for tumor localization prior to global attention, achieving AUC values approaching 0.999 on Kvasir (67). Swin-T-based approaches employing shifted window attention have demonstrated improved modeling of short- and long-range dependencies, reporting 95.4% accuracy on Kvasir v2 and 86.8% on HyperKvasir (68). Recurrent and multimodal transformer variants have further been introduced to refine temporal modeling and cross-modal fusion in gastrointestinal classification, with accuracies nearing 99% on HyperKvasir and related datasets (69, 70).

Comparative evaluations between CNN and transformer architectures have also produced mixed findings in gastrointestinal endoscopy. In anatomical site identification from ordinary and capsule gastroduodenoscopy images, CNN and ViT models were trained under identical optimization settings for image quality assessment and anatomical region classification. Although transformer-based models demonstrated competitive performance, CNN architectures achieved superior prospective generalization and overall classification performance across several evaluation settings, suggesting that purely transformer-based formulations may not consistently outperform convolutional models in gastrointestinal imaging tasks (71).

Several studies have proposed explicit CNN–Transformer hybrids to integrate fine-grained mucosal texture modeling with global contextual reasoning in gastrointestinal imaging. EfficientViT combines EfficientNet-B0 for local feature extraction with a ViT encoder for global dependency modeling through a dual-block structure, where one branch processes convolutional features and the other encodes global attention. Using fivefold cross-validation on eight gastrointestinal disease classes, accuracies up to 99.82% were reported, marginally exceeding MobileNetV2 baselines (64).

Rebba and Kamepalli (72) integrated RegNet with a Swin-T to enhance contextual interpretation of endoscopic images, benchmarking additional combinations such as MobileNetV2–Swin and ResNet152–ViT; the RegNet–Swin configuration achieved 90% accuracy. Ganesh et al. (73) introduced a computationally efficient CNN–Transformer hybrid emphasizing reduced parameter count and faster convergence, reporting 94% classification accuracy while lowering storage and training cost relative to conventional pretrained backbones. Hossain et al. (74) employed a hybrid transfer learning strategy in which Xception-like convolutional features were forwarded to a Swin-based transformer classifier, achieving 87.23% accuracy and outperforming Compact Convolutional Transformer and External Attention Transformer baselines. Subedi et al. (75) fused DenseNet201 with a Swin-T and demonstrated improved robustness under class imbalance on GastroVision and Kvasir-Capsule datasets, reporting competitive precision, recall, F1-score, and MCC metrics along with saliency-based interpretability.

Beyond endoscopic still images, hybrid transformer frameworks have also been applied to histopathology and capsule imaging. GasHis-Transformer integrates a position-encoded transformer branch with convolutional local feature extraction for gastric histopathological image detection, incorporating DropConnect regularization to reduce model size while maintaining stable performance (76). FLATer combines residual convolutional blocks, a ViT module, and spatial attention to jointly model local and global features, achieving 96.4% binary and 99.7% ternary classification accuracy with high computational throughput, even when trained from scratch (77). In wireless capsule endoscopy, segmentation-enhanced transformer architectures have been proposed; Özba (78) incorporated split-token embeddings and cross-channel feature learning within a transformer framework to reach 99.50% accuracy on the WCECCD dataset, while Bai et al. adopted a spatial pooling-enhanced ViT trained from scratch, achieving 79.15% multi-class accuracy on Kvasir-Capsule and 98.63% binary accuracy on the Red Lesion Endoscopy dataset (79).

Recurrent and ensemble-based transformer systems have further extended gastrointestinal analysis. Abuhayi (69) combined wavelet-based preprocessing with a concatenated recurrent ViT and an ensemble of classical classifiers, achieving 99.13% accuracy and AUC of 0.9954 on HyperKvasir. Sharmila and Geetha (70) proposed a multimodal recurrent transformer integrating clinical text (Bio-RoBERTa) and WCE imagery via cross-attention and ensemble decision modules, reporting 99.82% accuracy and strong interpretability across six gastrointestinal diseases. Tang et al. (7) introduced TransMT-Net, a multi-task transformer framework jointly optimizing classification and segmentation, achieving 96.94% classification accuracy and 77.76% Dice coefficient, with active learning mitigating limited labeled data. Suhasini et al. (80) developed RIViT, integrating residual, inception, and transformer encoder modules within a unified architecture, achieving 97% multi-class accuracy on WCE images. Zhang et al. designed a transformer-based detection architecture with a lightweight detection head and simplified feature pyramid network, demonstrating state-of-the-art F1, precision, and recall on gastroscopy and colonoscopy images (81).

Pure transformer approaches have also been investigated for gastrointestinal disease classification. Hosain et al. (66) applied a ViT with transfer learning to WCE colon images, reporting 95.63% accuracy and outperforming DenseNet201. Kakarla et al. (82) evaluated a ViT model on the 27-class GastroVision dataset, achieving 85.57% F1-score and outperforming DenseNet-121. Mansour et al. (83) proposed an attention-based hybrid deep learning framework integrating ViT, EfficientNetB7, ShuffleNetV2, and a convolutional bi-LSTM classifier, reporting improved performance over existing methods through feature engineering and attention-guided fusion.

### 2.5 Explainable AI in gastrointestinal imaging

The growing deployment of deep learning in gastrointestinal imaging has heightened concerns about interpretability, clinical accountability, and regulatory compliance. In digestive endoscopy, where diagnostic decisions directly influence biopsy, resection, or surveillance strategies, opaque “black-box” predictions remain a barrier to clinical adoption. XAI has therefore been introduced to improve transparency, support traceability requirements (e.g., GDPR-related accountability), and reinforce clinician trust in AI-assisted workflows (84, 85). In practice, most gastrointestinal-focused XAI implementations rely on visual heatmaps or feature-attribution techniques layered on top of classification models.

Saliency-based visual explanations remain the dominant paradigm. Grad-CAM, Grad-CAM++, guided Grad-CAM, and related heatmap techniques have been widely applied to highlight regions driving model predictions in endoscopic images (86–89). In hybrid Swin–CNN and lightweight CNN-based gastrointestinal classifiers, Grad-CAM visualizations have been used to verify that attention maps correspond to mucosal abnormalities, thereby reducing false-negative risk and increasing diagnostic confidence (88, 90, 91). Mukhtorov et al. (89) combined ResNet152 with Grad-CAM on the Kvasir dataset and demonstrated high classification performance alongside visually interpretable activation maps. Masum et al. (86) evaluated ViT, Swin-T, and an ensemble transformer model for classifying GERD, polyp, and normal categories, incorporating Grad-CAM and Grad-CAM++ to provide lesion-focused visual explanations and reporting 87% overall accuracy with balanced F1 scores. TeleXGI integrated ResNet50 and MobileNetV2 with Grad-CAM, saliency maps, integrated gradients, attribution heatmaps, and LIME, achieving diagnostic accuracies above 98% while validating explanation maps against Kvasir-SEG ground-truth polyp masks (87). Swin-based hybrid frameworks have similarly embedded Grad-CAM to reduce false negatives and provide class-specific visual rationale in gastrointestinal classification tasks (90, 92).

Beyond heatmaps, concept- and feature-based explanations have also been explored. Concept-driven XAI tools evaluated with gastroenterologists in polyp and tumor detection contexts were considered informative for model developers but not yet sufficient for routine clinical decision-making, highlighting limitations in user interface design and concept alignment with clinical reasoning (93). Feature attribution approaches integrating dimensionality reduction (e.g., PCA) and SHAP-based importance analysis have been coupled with gastrointestinal classifiers to expose influential learned features across multi-stage pipelines (91). Ahamed et al. (88) employed SHAP, guided heatmaps, Grad-CAM, and saliency mapping within a lightweight PD-CNN–PCC–EELM framework for multi-class gastrointestinal classification, achieving strong ROC-AUC performance while emphasizing interpretability and computational efficiency. Multimodal transformer systems integrating CNNs, RNNs, and transformers have likewise incorporated Grad-CAM, SHAP, and LIME to present explanatory evidence alongside predictions, aiming to enhance clinician confidence in automated gastrointestinal disease identification (94).

### 2.6 Research gap and scope

The existing body of work in gastrointestinal image analysis demonstrates two parallel trajectories: increasingly powerful hybrid local–global architectures and widespread use of *post-hoc* explainability tools. However, these developments have largely progressed independently and often without systematic architectural control or rigorous validation.

First, although CNN–Transformer hybrids and hierarchical transformer models consistently show performance gains, their fusion strategies are typically predefined in a structural manner. Local and global features are merged through concatenation, addition, cross-attention blocks, or stage-wise integration embedded within the backbone. In most cases, the contribution of each branch is implicitly learned through shared weights rather than explicitly regulated. This becomes a critical limitation in gastrointestinal imaging, where disease presentation varies markedly—ranging from diffuse inflammatory patterns requiring global reasoning to sharply localized lesions demanding fine-grained spatial discrimination. Fixed or loosely coupled fusion mechanisms may therefore fail to optimally balance contextual reasoning and structural detail on a per-sample basis. Explicit, gated, sample-adaptive fusion within transformer-based gastrointestinal classification remains insufficiently explored.

Second, XAI has been widely applied in gastrointestinal studies, but primarily as isolated visual overlays. Most works employ a single explanation tool—commonly Grad-CAM—without evaluating explanation stability, cross-method consistency, or alignment with quantitative class-wise performance. Moreover, XAI is rarely examined alongside architectural ablation or statistical testing, which limits confidence in the interpretability of reported results. While interpretability is acknowledged as essential for clinical adoption, its assessment is often descriptive rather than systematic.

Third, methodological validation across many gastrointestinal transformer studies remains uneven. High classification accuracies are frequently reported, yet fewer works provide comprehensive ablation analyses, statistically grounded model comparisons, or detailed class-wise evaluations that clarify the contributions of architectural components, such as fusion design.

The present study addresses these gaps through a unified framework built on three pillars. Architecturally, a dual-branch transformer encoder—comprising ViT-L/32 for global token-level reasoning and MaxViT-L for hierarchical spatial modeling—is integrated through an explicit adaptive gated fusion mechanism. This gate learns to dynamically weight local and global representations for each input, enabling controlled cross-scale modulation aligned with lesion morphology, anatomical spread, and visual complexity. Methodologically, extensive ablation and statistical analyses are conducted to isolate the effect of backbone selection, fusion strategy, and optimization design. Finally, although explainability is implemented *post-hoc* rather than embedded within the training objective, multiple complementary XAI approaches are systematically applied to assess visual rationale, cross-class consistency, and decision stability.

By combining adaptive gated hybridization, rigorous quantitative validation, and multi-approach *post-hoc* explainability within a single classification framework, the study advances both architectural control and interpretive transparency in transformer-based gastrointestinal disease detection.

## 3 Dataset and data preparation

This section outlines the characteristics of the gastrointestinal endoscopic dataset and the data preparation procedures implemented before model training. Standardization, normalization, and controlled augmentation were applied to ensure compatibility with pretrained transformer backbones while preserving clinically relevant visual patterns.

### 3.1 Dataset description

The experiments were conducted using the publicly available Kvasir gastrointestinal image dataset, accessible via Kaggle (https://www.kaggle.com/datasets/meetnagadia/kvasir-dataset). The dataset contains endoscopic images representing eight clinically relevant gastrointestinal categories: Dyed-Lifted Polyps (DLP), Dyed-Resection Margins (DRM), Esophagitis (EP), Normal Cecum (NC), Normal Pylorus (NP), Normal Z-line (NZL), Polyps (PP), and Ulcerative Colitis (UC). These categories encompass both normal anatomical structures and pathological findings, enabling comprehensive multi-class disease discrimination.

**Figure 2:**
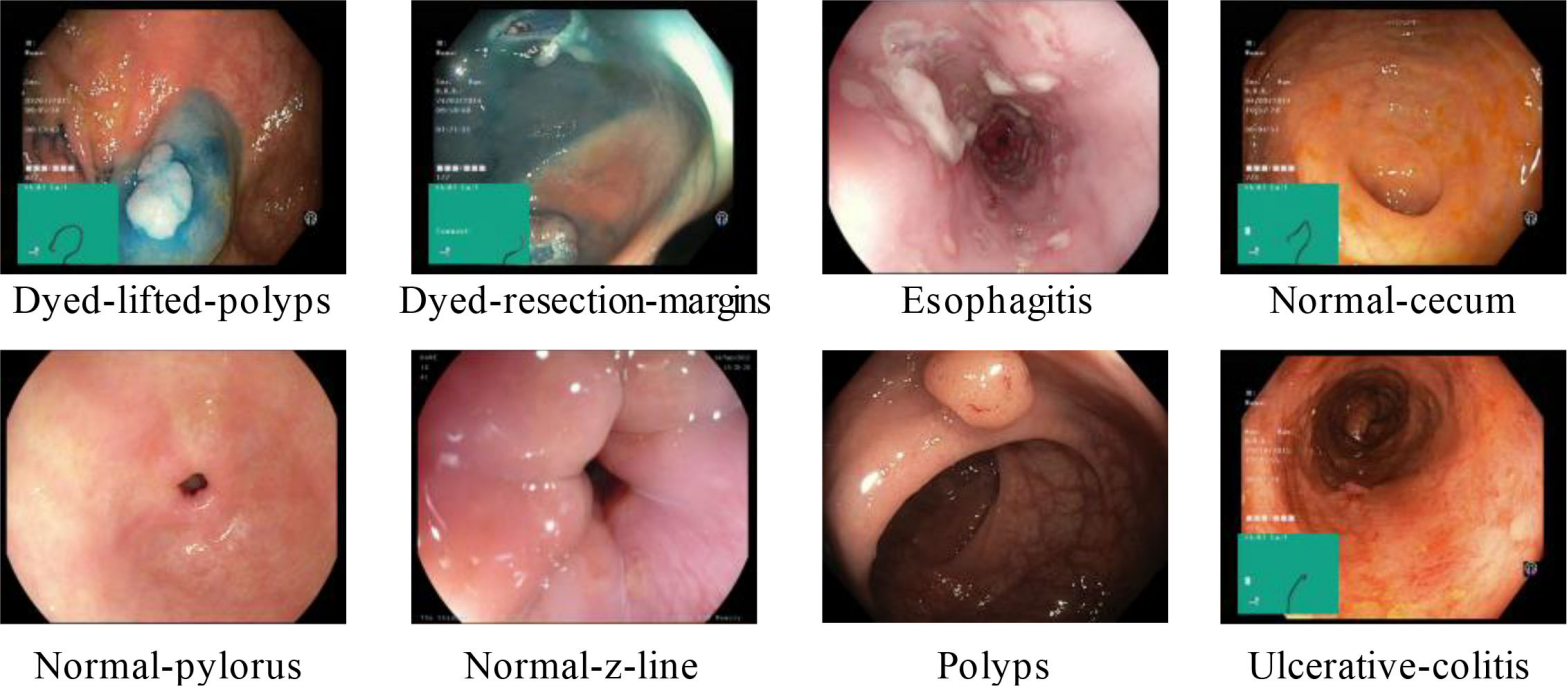
Representative endoscopic images from the eight gastrointestinal disease categories in the Kvasir dataset.

Each class contains 500 images, for a total of 4,000 endoscopic images. The images exhibit variations in illumination, mucosal texture, lesion morphology, and imaging angle, reflecting realistic clinical acquisition conditions. The inclusion of both inflammatory conditions (e.g., Esophagitis, Ulcerative Colitis) and structural lesions (e.g., Polyps, Dyed-Lifted Polyps) provides a balanced framework for evaluating fine-grained inter-class separability. Representative examples from each category are illustrated in **Figure 2**.

For experimental evaluation, the dataset was partitioned using a stratified split to preserve class distribution across subsets. Specifically, 70% of the images were allocated for training (n = 2,800), 10% for validation (n = 396), and 20% for independent testing (n = 804). The class-wise distribution across the three subsets is detailed in **Table 1**. Minor variations in validation and testing counts arise from integer rounding during stratified allocation.

**Table 1:** Stratified allocation of the Kvasir gastrointestinal dataset across experimental subsets.

Splitting sets	Dyed-lifted-polyps	Dyed-resection-margins	Esophagitis	Normal-cecum	Normal-pylorus	Normal-z-line	Polyps	Ulcerative-colitis	Total
Training (70%)	350	350	350	350	350	350	350	350	2800
Validation (10%)	50	49	50	49	49	49	50	50	396
Testing (20%)	100	101	100	101	101	101	100	100	804

The balanced nature of the dataset eliminates the need for class rebalancing techniques and ensures unbiased multi-class performance evaluation. This structure makes the dataset well-suited for assessing transformer-based architectures in robust gastrointestinal disease classification settings.

### 3.2 Image preprocessing

A standardized preprocessing pipeline was implemented to ensure compatibility with pretrained transformer backbones while preserving diagnostically relevant endoscopic features.

#### 3.2.1 Image resizing and standardization

All gastrointestinal endoscopic images were resized to 224 × 224 × 3 pixels to match the input resolution requirements of the pretrained ViT-L/32 and MaxViT-L architectures. Bicubic interpolation was employed during rescaling to maintain mucosal texture continuity and minimize structural distortion. Preserving fine-grained surface patterns is particularly important in endoscopic analysis, where subtle variations in vascularity, inflammation, and lesion morphology contribute to diagnostic discrimination.

#### 3.2.2 Pixel intensity normalization

To ensure numerical stability and compatibility with pretrained transformer backbones, pixel intensities were standardized prior to model input. Each RGB image was first scaled to the range [0,1] by dividing raw pixel values by 255.

Subsequently, channel-wise normalization was performed using the ImageNet mean and standard deviation statistics, consistent with the initialization of the pretrained ViT-L/32 and MaxViT-L models. The normalization was applied using Equation 1.

(1) $${\tilde{x}}_{c}=\frac{x_{c}-\mu_{c}}{\sigma_{c}},c\in \left\{R,G,B\right\}$$

where *x_c_* denotes the scaled pixel intensity of channel *c*, and ${\tilde{x}}_{c}$ represents the normalized output. The channel-wise mean and standard deviation values were: *μ* = (0.485, 0.456, 0.406) and σ = (0.229, 0.224, 0.225).

This transformation aligns the input distribution with the feature statistics learned during large-scale ImageNet pretraining, facilitating stable gradient propagation and accelerating convergence during fine-tuning. Channel-wise normalization also reduces illumination variability and inter-device intensity differences commonly encountered in endoscopic imaging, thereby improving robustness and generalization.

#### 3.2.3 Data augmentation strategy

To improve generalization and reduce overfitting, online data augmentation was applied exclusively to the training subset. The augmentation protocol simulated realistic procedural variability encountered during live endoscopy.

Geometric transformations included:

- Random horizontal flipping (probability = 0.5)
- Random rotation within ±15°
- Random affine translation up to 10%
- Random scaling within the range 0.9–1.1

These operations emulate changes in camera orientation, minor patient movement, and variations in scope positioning.

#### 3.2.4 Robustness to clinical artifacts

To enhance resilience against acquisition-related noise and motion artifacts, mild Gaussian noise and low-probability Gaussian blur were introduced during training. These perturbations approximate sensor noise, minor motion blur, and illumination fluctuations, thereby improving robustness under real-world clinical deployment conditions.

#### 3.2.5 Dataset partitioning and class balance

The dataset comprised uniformly distributed classes (500 images per category). Consequently, no oversampling or synthetic class balancing techniques were required. Stratified splitting was performed to preserve class proportions across subsets, with 60% allocated for training, 10% for validation, and 30% for independent testing. This ensured consistent distribution of disease categories during optimization and evaluation.

#### 3.2.6 Transformer-specific preparation

For the ViT-L/32 branch, each preprocessed image was partitioned into non-overlapping 32 × 32 patches, yielding 49 patch tokens per image before positional encoding and transformer processing. For the MaxViT-L branch, hierarchical feature representations were generated via an initial convolutional projection stage followed by multi-axis attention encoding, enabling the structured extraction of local and global spatial dependencies.

## 4 Proposed hybrid architecture

**Figure 3:**
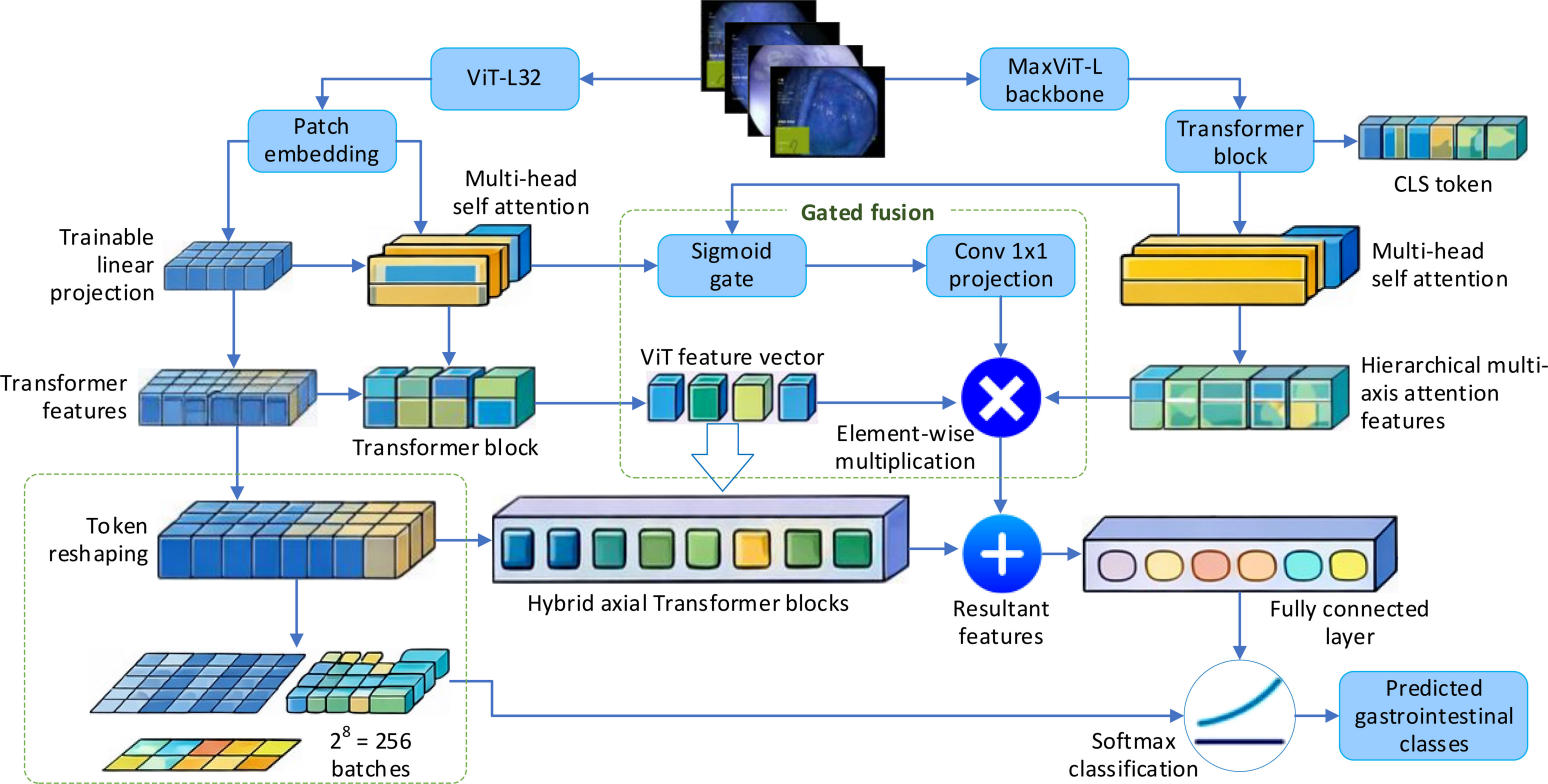
Proposed hybrid ViT–MaxViT architecture with adaptive gated fusion for gastrointestinal disease classification.

The proposed model is a two-branch hybrid transformer designed to jointly capture global semantic context and hierarchical spatial structure for multiclass gastrointestinal disease detection, as illustrated in **Figure 3**. The architecture integrates a token-based ViT (ViT-L/32) and a hierarchical MaxViT-L backbone, followed by projection into a shared latent space, adaptive gated fusion, and a lightweight classifier head. The ViT branch models long-range dependencies through global patch-token interactions, whereas the MaxViT branch captures multi-scale spatial structure via hierarchical multi-axis attention.

The design is motivated by representational complementarity. While global self-attention facilitates contextual reasoning across distant regions, it lacks strong locality bias; conversely, hierarchical architectures preserve spatial inductive structure but may constrain full-image contextual aggregation. By combining these paradigms within a unified framework and introducing a learnable gating mechanism, the model enables sample-specific weighting between global and local representations rather than relying on fixed fusion.

Both branches process the same RGB input image resized to 224×224. Their feature embeddings are aligned through projection into a common latent space, after which an adaptive gate regulates their relative contributions via element-wise fusion. The resulting representation is passed to a fully connected classification head to predict gastrointestinal disease categories. This formulation allows the model to emphasize fine-grained mucosal patterns or broader anatomical context depending on lesion characteristics and imaging variability.

### 4.1 ViT branch (global patch-token reasoning)

The first branch employs a ViT-L/32 backbone pretrained on ImageNet-21k and configured as a feature extractor. The input image is partitioned into non-overlapping 32×32 patches, producing 49 patch tokens. Each patch is flattened and linearly projected into a high-dimensional embedding space, and positional encodings are added to preserve spatial ordering.

The token sequence is processed by 24 stacked transformer encoder layers, each composed of multi-head self-attention (MHSA) and feed-forward sublayers. Self-attention enables each patch token to interact with all other tokens, allowing the model to capture long-range dependencies and global semantic coherence across the image.

The final classification token (CLS token) serves as the global image representation, defined by Equation 2:

(2) $$f_{\text{ViT}}\in {\mathbb{R}}^{1024}$$

This embedding encodes holistic contextual information, which is particularly beneficial for detecting diffuse inflammation, large-area mucosal changes, or distributed pathological patterns that require global reasoning rather than localized inspection.

The ViT branch thus functions as a global semantic encoder, providing context-aware features that complement the hierarchical spatial modeling of the second branch.

### 4.2 MaxViT branch (hierarchical multi-axis spatial modeling)

The second branch employs a MaxViT-Large backbone pretrained on ImageNet-21k and configured as a feature extractor. Unlike pure token-based processing in ViT, MaxViT integrates convolutional inductive bias with structured multi-axis attention, enabling simultaneous local detail preservation and long-range spatial interactions.

The input image is first processed by a convolutional stem that extracts low-level spatial features. The network then progresses through multiple hierarchical stages. Within each stage, two complementary attention mechanisms are applied:

- Window-based attention, which models fine-grained local spatial dependencies within fixed spatial regions.
- Grid-based attention, which captures structured long-range interactions across the feature map.

This multi-axis design preserves locality while avoiding the quadratic complexity of full global attention over all pixels. As the network deepens, spatial resolution is progressively reduced while channel dimensionality increases, producing semantically enriched feature maps.

At the final stage, global average pooling is applied to obtain a compact representation, as shown in Equation 3:

(3) $$f_{\mathrm{Max}\text{ViT}}\in {\mathbb{R}}^{D_{m}}$$

This embedding retains hierarchical spatial cues, including lesion boundaries, vascular texture, polyp morphology, and fine mucosal irregularities. These localized features are often critical in gastrointestinal disease classification, particularly when pathological indicators are subtle and spatially confined.

The MaxViT branch, therefore, functions as a hierarchical structural encoder, complementing the global semantic abstraction of the ViT branch.

### 4.3 Projection to a common latent space

Although both branches produce high-level embeddings, their representational characteristics and dimensionalities may differ. Direct fusion without alignment can introduce scale imbalance and representation mismatch.

To address this, both embeddings are linearly projected into a shared latent space of dimension *D_f_* (recommended *D_f_* = 1024), defined by Equations 4 and 5:

(4) $${\tilde{f}}_{\text{ViT}}=W_{v}f_{\text{ViT}}$$

(5) $${\tilde{f}}_{\text{MaxViT}}=W_{m}f_{\text{MaxViT}}$$

This projection serves two purposes:

1. Dimensional alignment – ensuring both branches contribute features of comparable size.
2. Representation calibration – enabling the fusion mechanism to operate within a unified embedding manifold.

By explicitly aligning the embeddings before fusion, the model avoids bias toward one branch due to dimensional dominance or scale discrepancy.

### 4.4 Adaptive gated fusion mechanism

Rather than employing static concatenation or uniform averaging, the proposed architecture utilizes a learnable gating mechanism to dynamically balance global and hierarchical features.

The projected embeddings are concatenated and passed through a lightweight multi-layer perceptron followed by a sigmoid activation, as shown in Equation 6:

(6) $$g=\sigma \left(W_{g}\left[{\tilde{f}}_{\text{ViT}};{\tilde{f}}_{\text{MaxViT}}\right]+b_{g}\right)$$

where g∈(0,1) is a sample-adaptive fusion coefficient.

The final fused representation is computed using Equation 7:

(7) $$f_{\text{fused}}=g\cdot {\tilde{f}}_{\text{ViT}}+\left(1-g\right)\cdot {\tilde{f}}_{\text{MaxViT}}$$

The gating formulation allows the model to modulate the relative contribution of global and hierarchical features in a sample-dependent manner. This adaptive weighting mitigates the limitations of static feature fusion and reduces representational redundancy between branches. By permitting differential emphasis on contextual semantics and localized structural cues, the fusion module enhances robustness to heterogeneous gastrointestinal disease patterns. The resulting fused embedding $f_{\text{fused}}\in {\mathbb{R}}^{D_{f}}$ is subsequently forwarded to the classifier head for final prediction.

### 4.5 Classifier head

The fused representation $f_{\text{fused}}\in {\mathbb{R}}^{D_{f}}$ is passed to a lightweight classification head to produce logits for the eight gastrointestinal disease categories.

The classifier consists of:

- Layer Normalization (optional but recommended for stability)
- Dropout for regularization
- A fully connected linear layer mapping *D_f_* → 8

The logits are computed as using Equation 8:

(8) $$o=W_{c}f_{\text{fused}}+b_{c}$$

where $W_{c}\in {\mathbb{R}}^{8\times D_{f}}$.

The final class probabilities are obtained using the softmax function. A detailed summary of the architectural configuration of the proposed hybrid transformer is provided in **Table 2**.

**Table 2:** Backbone specifications and fusion design of the proposed hybrid transformer.

Module	Specification	Output shape (batch = B)
Input	RGB endoscopy image	(B, 3, 224, 224)
Branch-1 encoder	ViT-L/32 feature extractor (num_classes=0)	(B, 1024) pooled, or (B, 50, 1024) tokens
Branch-2 encoder	MaxViT-Large feature extractor (num_classes=0)	(B, 1024) pooled, or (B, 1024, 7, 7) map
Projection	Linear projection to common fusion dim Df (e.g., 1024)	(B, Df) + (B, Df)
Fusion	Gated weighted sum $g=\sigma \left(W_{2}\;\phi \left(W_{1}\left[f_{\text{vit}};f_{\text{max}}\right]+b_{1}\right)+b_{2}\right)$ where, fvit: feature vector from the ViT branch, fmax feature vector (e.g., max-pooled CNN features), [fvit; fmax]: concatenation of the two feature vectors, W1, W2: learnable weight matrices of the MLP, b1, b2: bias terms, ϕ(·): nonlinear activation (GELU, etc.), σ(·): Sigmoid function ensuring g∈(0,1), g: learned gating vector (or scalar) controlling feature contribution	(B, Df)
Classifier head	LayerNorm → Dropout → Linear	(B, 8)
Loss	Cross-Entropy	scalar

## 5 Mathematical formulation of the proposed hybrid transformer and optimization

This section formalizes the proposed hybrid transformer architecture described in Section 3. The formulation integrates the ViT-L/32 branch, MaxViT-L branch, projection alignment, adaptive gated fusion, and classification module into a unified trainable framework.

### 5.1 Formal model definition

Let, D (defined by Equation 9) denote the gastrointestinal endoscopy dataset, where $x_{i}\in {\mathbb{R}}^{224\times 224\times 3}$ represents an RGB image resized to 224×224, and $y_{i}\in \left\{1,\;2,\ldots ,\;8\right\}$ denotes the corresponding disease label.

(9) $$D={\left\{\left(x_{i},y_{i}\right)\right\}}_{i=1}^{N}$$

#### 5.1.1 ViT-L/32 global representation learning

Each input image *x* is divided into non-overlapping patches of size *P*×*P*, where *P* = 32. The number of patches is calculated using Equation 10.

(10) $$N_{p}={\left(\frac{224}{32}\right)}^{2}=49$$

Each patch $x_{i}^{p}\in {\mathbb{R}}^{32\times 32\times 3}$ is flattened and linearly projected into a D-dimensional embedding space using Equation 11:

(11) $$z_{i}=E\cdot \text{vec}\left(x_{i}^{p}\right)+b,i=1,\ldots ,\;49$$

where $E\in {\mathbb{R}}^{D\times \left(P^{2}\cdot 3\right)}$. This projection transforms spatial patches into token embeddings suitable for sequence modeling within the transformer encoder.

A learnable classification token is prepended to the patch sequence, and positional embeddings are added to preserve spatial order through Equation 12:

(12) $$Z^{\left(0\right)}=\left[z_{\text{cls}};z_{1};\ldots \;;z_{49}\right]+E_{\text{pos}}$$

The Transformer encoder contains *L* = 24 stacked blocks. At each layer through Equations 13 and 14:

(13) $${\hat{Z}}^{\left(l\right)}=Z^{\left(l-1\right)}+\text{MHSA}\left(\text{LN}\left(Z^{\left(l-1\right)}\right)\right)$$

(14) $$Z^{\left(l\right)}={\hat{Z}}^{\left(l\right)}+\text{FFN}\left(\text{LN}\left({\hat{Z}}^{\left(l\right)}\right)\right)$$

These residual connections preserve gradient flow, while multi-head self-attention models long-range dependencies among patch tokens.

For each attention head *h*, the scaled dot-product attention is defined as in Equation 15:

(15) $$\text{Attn}\left(Q_{h},K_{h},V_{h}\right)=\text{softmax}\left(\frac{Q_{h}K_{h}^{⊤}}{\sqrt{d_{h}}}\right)V_{h}$$

The final global image representation is obtained from the classification token, defined in Equation 16:

(16) $$f_{\text{ViT}}=Z_{\text{cls}}^{\left(L\right)}$$

This embedding captures holistic contextual information across the entire endoscopic image.

#### 5.1.2 MaxViT-L hierarchical multi-axis encoding

In parallel, the same input image is processed through the MaxViT-L branch, which combines convolutional inductive bias with structured multi-axis attention.

At stage *s*, feature maps are updated as using Equation 17:

(17) $$F^{\left(s\right)}=\phi^{\left(s\right)}\left(F^{\left(s-1\right)}\right)$$

where $\phi^{\left(s\right)}\left(\cdot \right)$ represents convolutional projection followed by attention operations.

Specifically, each stage applies window-based local attention followed by grid-based global attention (Equation 18):

(18) $$F^{\left(s\right)}=\text{GridAttn}\left(\text{WinAttn}\left(F^{\left(s-1\right)}\right)\right)$$

This multi-axis design enables simultaneous modeling of fine-grained spatial details and long-range structured interactions.

After the final stage *S*, global average pooling yields a compact hierarchical embedding as shown in Equation 19:

(19) $$f_{\text{MaxViT}}=\text{GAP}\left(F^{\left(S\right)}\right)$$

#### 5.1.3 Projection into shared latent space

To enable stable and balanced fusion, both branch outputs are projected into a shared latent space of dimension *D_f_*, as defined by Equations 20 and 21:

(20) $${\tilde{f}}_{\text{ViT}}=W_{v}f_{\text{ViT}}+b_{v}$$

(21) $${\tilde{f}}_{\text{MaxViT}}=W_{m}f_{\text{MaxViT}}+b_{m}$$

This projection aligns the statistical distributions and dimensionality of both embeddings prior to fusion.

#### 5.1.4 Adaptive gated fusion

A sample-adaptive fusion gate is defined as by Equation 22:

(22) $$g=\sigma \left(W_{g}\left[\begin{array}{c} {\tilde{f}}_{\text{ViT}}\\ {\tilde{f}}_{\text{MaxViT}} \end{array}\right]+b_{g}\right)$$

where g∈(0,1) controls the relative contribution of each branch.

The fused representation is computed as using Equation 25:

(23) $$f_{\text{fused}}=g\cdot {\tilde{f}}_{\text{ViT}}+\left(1-g\right)\cdot {\tilde{f}}_{\text{MaxViT}}$$

This formulation enables dynamic, sample-dependent weighting between global contextual reasoning and hierarchical spatial encoding.

#### 5.1.5 Classification

The fused feature vector is mapped to class logits using Equation 24:

(24) $$o=W_{c}f_{\text{fused}}+b_{c}$$

and class probabilities are obtained via SoftMax Equation 25:

(25) $${\hat{y}}_{k}=\frac{\exp\left(o_{k}\right)}{\sum_{j=1}^{8}\exp(o_{j})}$$

### 5.2 Training objective

The model is trained by minimizing categorical cross-entropy loss as defined in Equation 26:

(26) $$ℒ=-\frac{1}{N}\sum_{i=1}^{N}\sum_{k=1}^{8}I(y_{i}=k)\text{log}\left({\hat{y}}_{i,k}\right)$$

The optimal parameters are obtained as using Equation 27:

(27) $${\text{Θ}}^{*}=\text{arg}\;\underset{\text{Θ}}{\text{min}}\;ℒ\left(\text{Θ}\right)$$

The objective in Equation 27 is minimized with respect to Θ using stochastic gradient-based backpropagation, enabling end-to-end optimization of both backbone branches and the adaptive fusion module. The complete training procedure is summarized in Algorithm 1.

Algorithm 1

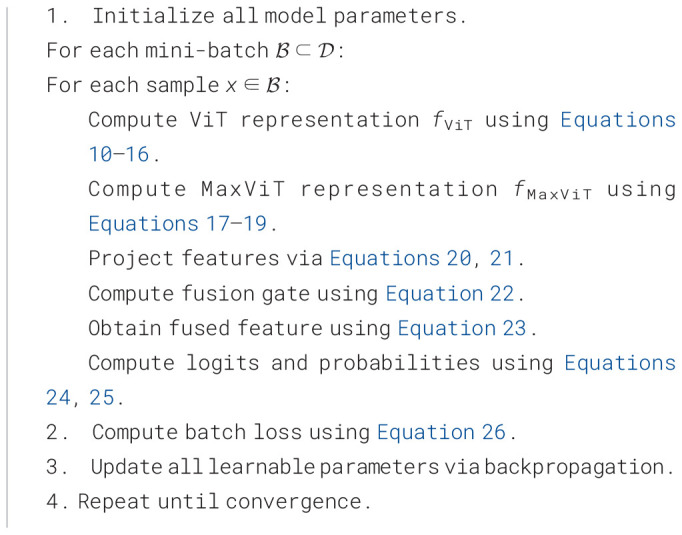

## 6 Experimental results

All experiments were conducted on a high-performance Dell workstation equipped with an Intel^®^ Core™ i9-10900K CPU (3.70 GHz), an NVIDIA GeForce RTX 2080 Ti GPU (11 GB GDDR6), 64 GB DDR4 RAM, a 500 GB NVMe SSD, and a 2 TB HDD, running Windows 11 Pro. Despite the computational complexity of the proposed hybrid transformer architecture, the available hardware configuration was sufficient to support end-to-end training using pretrained backbones, controlled fine-tuning, and optimized batch-wise training. Implementation was carried out using Python within the Anaconda–Jupyter Notebook environment. The training hyperparameters and optimization settings used in all experiments are summarized in **Tables 3**, **4**.

**Table 3:** Hyperparameters and their values.

Hyperparameters	Values
Optimizer	Adam
Learning rate	0.001
Activation	Softmax
Loss function	Cross-entropy (categorical)
Dropout probability	0.3
Batch size	32
Epochs	100

**Table 4:** Training configuration and optimization settings.

Block	Layer/operation	Output shape (B=batch)	Key hyperparameters
Input	RGB endoscopy image	(B, 3, 224, 224)	—
Branch-A encoder	ViT-Large/32 backbone (feature extractor)	(B, Dv)	Patch=32, pretrained ImageNet-21k (Hugging Face)
Branch-B encoder	MaxViT-Large backbone (feature extractor)	(B, Dm)	Multi-axis attention, pretrained ImageNet-21k (Hugging Face)
Projection-A	Linear: Dv → Df	(B, Df)	Df = 1024 (recommended)
Projection-B	Linear: Dm → Df	(B, Df)	Df = 1024 (recommended)
Fusion (gated)	g = σ(MLP([fA; fB]))	(B, 1)	MLP: 2Df → Df → 1
Fused embedding	f = g·fA + (1−g)·fB	(B, Df)	Per-sample adaptive weighting
Classifier head	LayerNorm → Dropout → Linear(Df→8)	(B, 8)	Dropout 0.2–0.5
Output	Softmax probabilities	(B, 8)	Cross-entropy (optionally label smoothing)

### 6.1 Training convergence and optimization behavior

**Figure 4:**
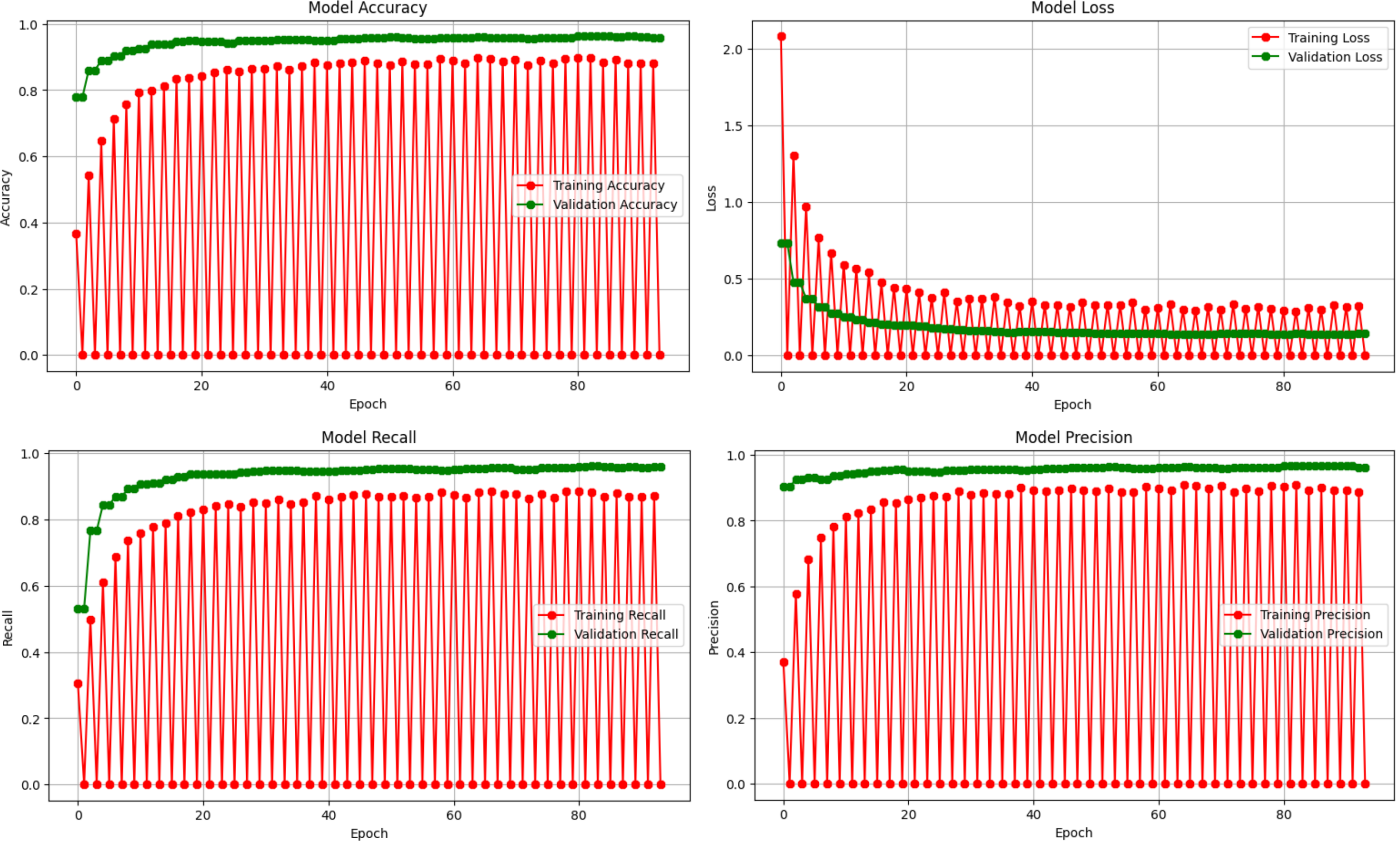
Convergence behavior of the proposed hybrid transformer showing training and validation accuracy, loss, recall, and precision across 100 epochs.

**Figure 4** illustrates the training and validation curves for accuracy, loss, recall, and precision over 100 epochs. The learning curves demonstrate stable convergence of the proposed hybrid transformer model. Training accuracy increases rapidly during the initial epochs and then progressively stabilizes, while validation accuracy closely tracks the same trend, plateauing above 0.95 after early convergence. Validation performance remains consistently aligned with training behavior throughout the optimization process.

Training loss exhibits a sharp decline in the early phase, followed by gradual stabilization. Validation loss decreases smoothly and remains consistently lower than training loss in later epochs, without divergence. The absence of late-epoch degradation or widening performance gaps indicates controlled optimization dynamics. Similarly, recall and precision curves show progressive improvement in early epochs, followed by a stable plateau. Validation recall stabilizes above 0.95, and validation precision reaches comparably high levels with minimal fluctuation. Training and validation recall–precision curves remain closely aligned, reflecting balanced classification performance across epochs.

### 6.2 Overall quantitative performance metrics

The proposed hybrid ViT-L/32 + MaxViT-L (Gated Fusion) model achieved an overall classification accuracy of 96.38%, demonstrating reliable multi-class discrimination across the eight gastrointestinal categories. The model achieved 96.02% precision and 95.74% recall, yielding an F1-score of 95.88%, indicating balanced performance between false-positive and false-negative predictions.

**Figure 5:**
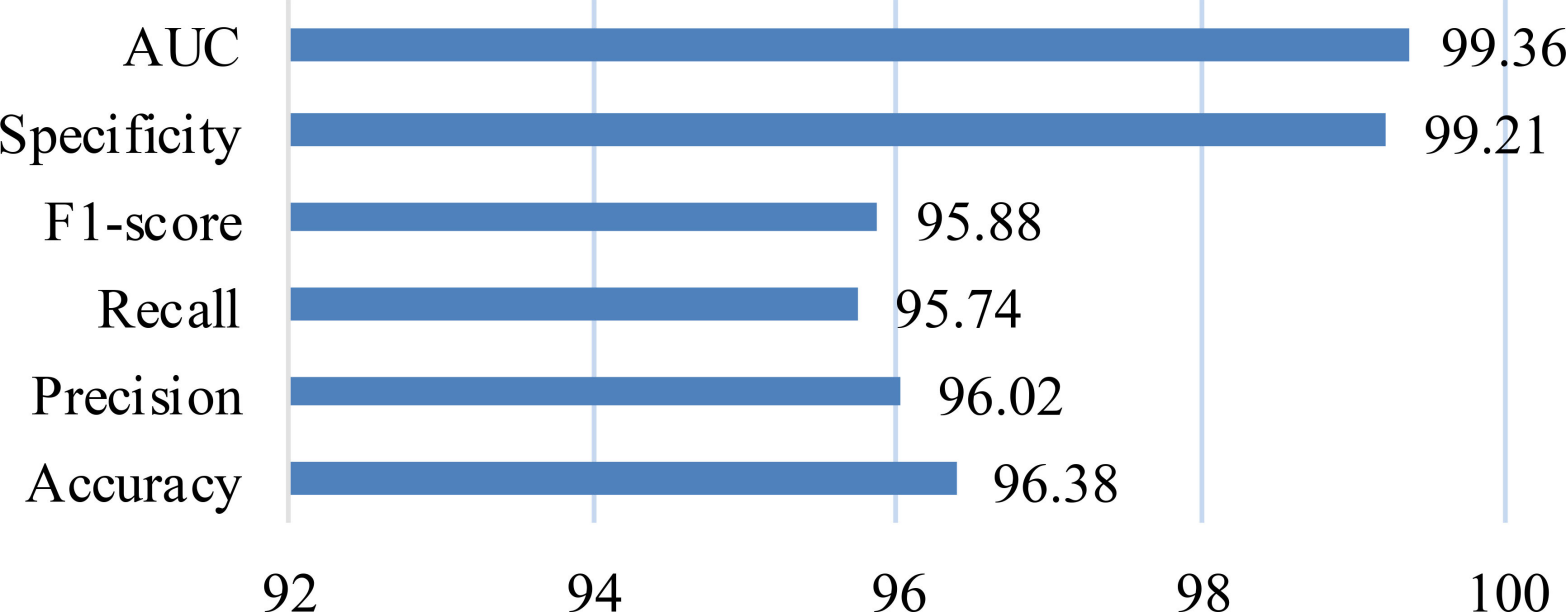
Overall quantitative performance metrics of the proposed hybrid model.

A specificity of 99.21% was observed, reflecting strong true-negative identification and clear separation between normal anatomical structures and pathological findings. The overall AUC reached 0.9936, confirming high separability across decision thresholds and stable ranking performance. The quantitative performance distribution across evaluation metrics is illustrated in **Figure 5**.

### 6.3 Class-wise discrimination performance

**Figure 6:**
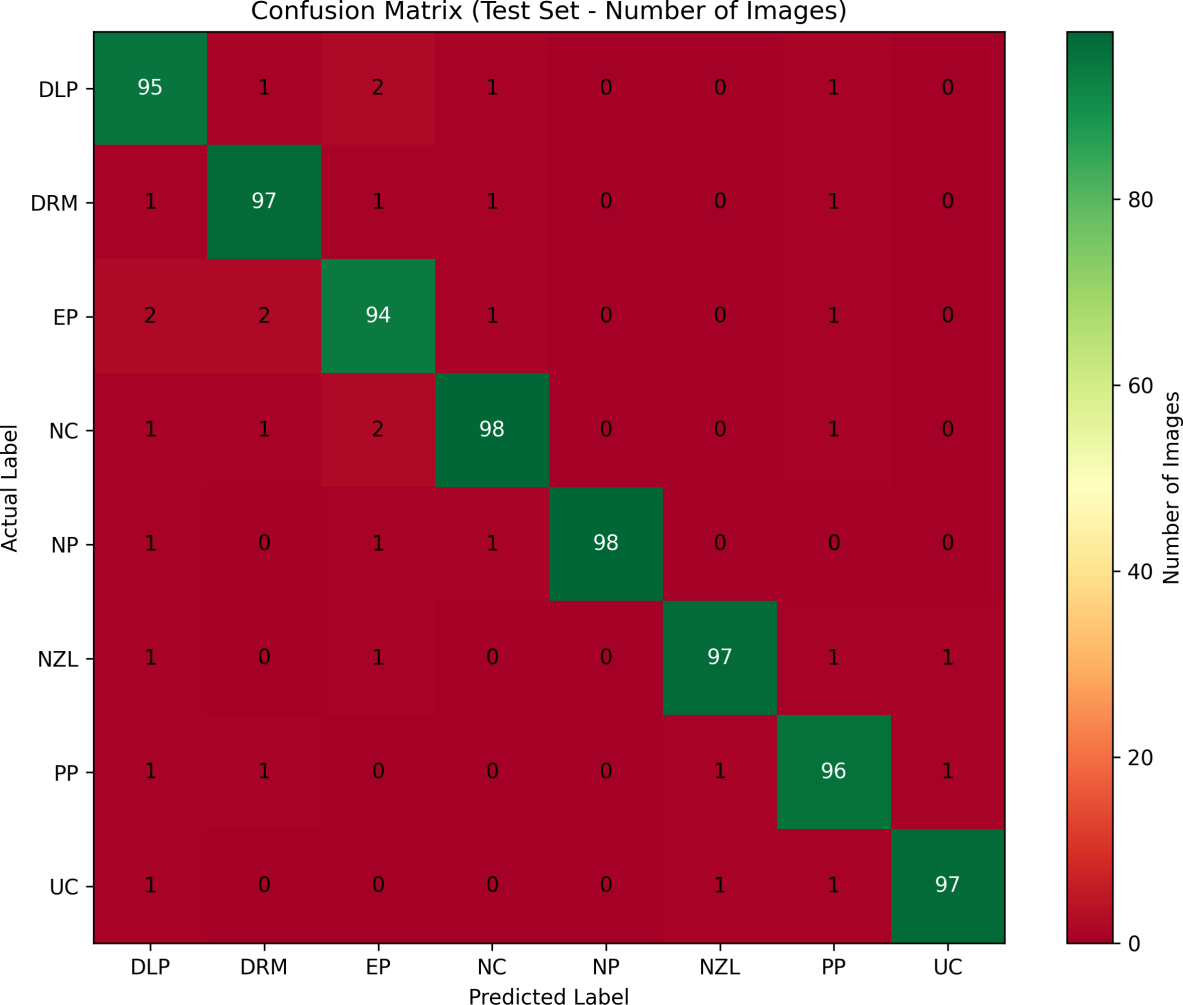
Confusion matrix of the proposed hybrid transformer for 8-class gastrointestinal disease classification.

**Figure 6** presents the confusion matrix for the eight-class gastrointestinal disease classification task. The confusion matrix demonstrates strong discriminative performance across all categories, with dominant diagonal elements indicating high class-wise accuracy. Correct predictions range from 94 to 98 samples per class. The Normal Cecum (NC) and Non-Polyp (NP) classes exhibit particularly strong separability, with 98 correctly classified instances each. Misclassifications are limited and typically involve one or two samples per class, without any systematic collapse into dominant categories.

Minor confusion is observed between visually related categories, such as EP and DLP or DRM, reflecting subtle morphological overlap in certain endoscopic presentations. Overall, off-diagonal dispersion remains minimal, indicating stable multi-class discrimination.

### 6.4 Receiver Operating Characteristic analysis

**Figure 7:**
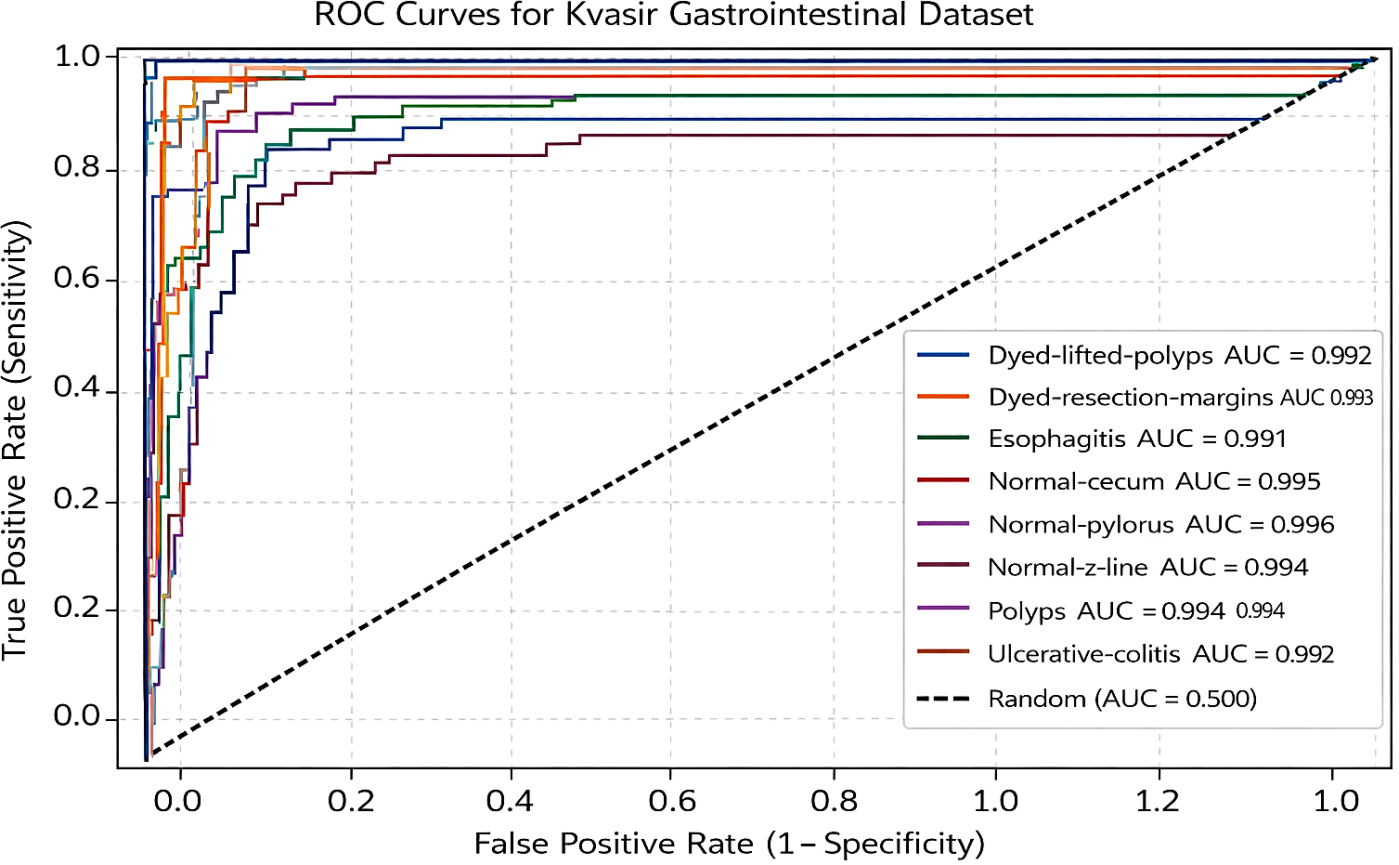
Class-wise ROC curves of the proposed hybrid transformer with corresponding AUC values.

**Figure 7** depicts the ROC curves for all eight gastrointestinal disease classes. The ROC curves demonstrate strong class separability, with AUC values ranging from 0.991 to 0.996. Normal-pylorus (AUC = 0.996) and Normal-cecum (AUC = 0.995) achieve the highest discrimination, while inflammatory and lesion-based categories, including Esophagitis (AUC = 0.991), Dyed-lifted polyps (AUC = 0.992), and Ulcerative colitis (AUC = 0.992), also show consistently high AUC values. All curves remain substantially above the random classifier baseline (AUC = 0.5), with steep initial rises indicating high sensitivity at low false positive rates.

### 6.5 Quantitative class-wise metrics

**Table 5** summarizes the detailed class-wise performance of the proposed hybrid transformer model. Precision values range from 95.64% to 98.68%, recall from 95.19% to 97.05%, and F1-scores from 95.41% to 97.35%. Specificity remains uniformly high across all classes (≥ 99.08%). Normal-pylorus achieves the highest F1-score (97.35%) and precision (98.68%), followed closely by Normal-cecum (F1 = 96.99%). Inflammatory and lesion-related classes also maintain balanced precision–recall profiles exceeding 95%. The macro-average performance further confirms consistency, with precision of 96.47%, recall of 96.05%, F1-score of 96.26%, specificity of 99.28%, and AUC of 0.9936, indicating stable multi-class behavior without class imbalance dominance.

**Table 5:** Class-wise performance of the proposed hybrid transformer model.

Gastrointestinal class	Precision	Recall	F1-score	Specificity	AUC
Dyed-lifted-polyps	95.82	95.41	95.61	99.14	99.24
Dyed-resection-margins	96.27	95.88	96.07	99.22	99.32
Esophagitis	95.64	95.19	95.41	99.08	99.1
Normal-cecum	97.18	96.81	96.99	99.43	99.51
Normal-pylorus	97.46	97.05	97.25	99.51	99.69
Normal-z-line	96.88	96.42	96.65	99.37	99.44
Polyps	96.54	96.11	96.32	99.29	99.4
Ulcerative-colitis	95.97	95.53	95.75	99.17	99.2
Macro average	96.47	96.05	96.26	99.28	99.36

### 6.6 Comparison with transformer-based architectures

**Table 6** presents a comparative evaluation of the proposed hybrid transformer against a diverse set of transformer-based architectures, including conventional ViTs, hierarchical transformers, medical-domain transformer variants, and recent hybrid attention-based models. Color-scale formatting is employed to facilitate rapid visual identification of relative performance differences across metrics.

**Table 6:** Comparative performance analysis of the proposed hybrid transformer and baseline transformer models.

Model	Accuracy	Precision	Recall	F1-score	Specificity	AUC
ViT-B/16	91.24	90.88	90.41	90.64	97.12	96.42
ViT-L/32	92.73	92.11	91.86	91.98	97.89	97.1
DeiT-Base	90.95	90.22	89.87	90.04	96.85	95.99
Swin-T-Base	93.84	93.27	92.91	93.09	98.34	97.81
TransUNet	92.48	91.93	91.54	91.73	97.63	96.97
MaxViT-Large	94.26	93.88	93.51	93.69	98.71	98.14
MedViT	93.12	92.56	92.03	92.29	98.02	97.53
TransFuse	92.94	92.31	91.98	92.14	97.88	97.2
Swin-UNETR	94.01	93.54	93.17	93.35	98.55	97.9
Pyramid ViT	93.45	92.97	92.58	92.77	98.21	97.6
FLATer	93.72	93.18	92.84	93.01	98.42	97.86
TransMT-Net	93.41	92.96	92.61	92.78	98.31	97.64
ReViT	92.89	92.44	92.17	92.3	98.12	97.22
Proposed hybrid transformer	96.38	96.02	95.74	95.88	99.21	99.36

Color shading stands for standard heatmap color representation.

The proposed ViT-L/32 + MaxViT-L (gated fusion) framework consistently achieves the strongest performance across all evaluation measures. Compared with standalone ViT-L/32 and MaxViT-Large models, the results suggest that combining global contextual modeling with multi-scale local feature extraction yields more discriminative representations. The proposed framework also outperforms recent transformer architectures such as FLATer, TransMT-Net, and ReViT, indicating that adaptive fusion of complementary feature spaces is more effective than relying solely on enhanced attention mechanisms or transformer-specific architectural refinements. Similar observations can be made against MedViT, TransFuse, and Swin-UNETR, where the proposed model maintains a favorable balance between sensitivity-oriented and specificity-oriented metrics. The consistently strong recall and specificity further suggest an improved ability to detect pathological regions while limiting false-positive predictions.

### 6.7 Comparison with state-of-the-art

**Table 7** presents a comparative analysis of recent transformer-based and hybrid approaches for gastrointestinal disease classification across commonly used datasets. Most prior studies report strong performance, particularly for hybrid CNN–Transformer models, with accuracies generally ranging from 87% to 95%. However, the reporting remains inconsistent, with several works lacking complete evaluation metrics such as specificity and AUC, and performance often varying with dataset size, class complexity, and split configuration.

**Table 7:** Comparison with state-of-the-art methods for gastrointestinal disease classification.

Ref.	Dataset used	No of images	No. of classes	Train: [validation]:test	Model with best accuracy	Accuracy (%)	Precision (%)	Recall (%)	F1-score (%)	Specificity (%)	AUC (%)	XAI Post-hoc
(66)	WCE (Wireless Capsule Endoscopy) curated colon dataset (Kaggle)	2000	4	80:0:20	ViT	95.63	91.75	89.25	88.75	–	–	×
(68)	Kvasir dataset (v2)	6000	6	60:20:20	Hybrid Shifted Window Transformer (HSW)	95.42	–	–	95.41	–	–	×
(72)	Kvasir dataset (Kaggle)	8000	8	80:0:20	RegNet + Swin-T (proposed hybrid model)	90.00	91.25	90.00	90.00	–	–	×
(73)	Kvasir dataset (v1)	8000	8	–	Proposed hybrid CNN + ViT-based attention layer	94.00	93.78	93.91	94.00	–	–	×
(74)	Kvasir-based gastrointestinal dataset (Kaggle)	3500	7	80:10:10	Hybrid Swin-T + Xception (proposed model)	87.23	87.30	85.40	85.30	–	94.00	×
(79)	Kvasir-capsule dataset and red lesion endoscopy (RLE) dataset	34338 and 3285	11 and 2	80:0:20	Proposed ViT with Spatial Pooling (ViT-based)	79.15(Kvasir-Capsule) 98.63 (RLE)	–	–	–	–	–	×
(82)	Kvasir dataset (GI endoscopy images)	8600	27	–	ViT	84.10	86.41	85.75	85.57	–	88.71	×
(86)	GastroEndoNet collected from Zainul Haque Sikder Women’s Medical College & Hospital	4006	4	80:10:10	Ensemble Transformer (ViT + Swin-T)	87.00	86.25	87.00	86.50	–	–	√
(88)	GastroVision dataset	8000	27	90:0:10	PD-CNN + PCC + EELM (proposed model)	87.75	88.12	87.75	87.12	–	98.89	√
(89)	Kvasir dataset	8000	8	80:10:10	ResNet-152 + Grad-CAM (Proposed XAI model)	93.46	–	–	–	–	–	√
(90)	Kvasir dataset	8000	8	70:0:30	Hybrid model: Swin-T + EfficientNet-B3 + ResNet50 + Stacked ML classifiers (XGBoost, LightGBM, SVM)	93.79	94.64	93.79	93.91	–	–	√
(92)	GastroVision dataset	8000	27	–	FGVC (Fine-grained visual classification) with Swin-T	83.00	82.80	83.00	82.50	81.00	88.12	√
(94)	Kvasir dataset + GastroNet dataset	–	–		Ensemble model (CNN + RNN + Transformer)	92.60	91.80	91.20	91.50	90.26	94.30	√
This paper	Kvasir gastrointestinal image dataset	4000	8	70:10:20	Proposed hybrid transformer	96.38	96.02	95.74	95.88	99.21	99.36	√

The proposed model achieves the highest overall performance, with an accuracy of 96.38%, along with consistently strong precision (96.02%), recall (95.74%), and F1-score (95.88%). Notably, it also reports specificity (99.21%) and AUC (99.36%), which are absent in many prior studies but are critical for clinical reliability.

In addition, while a subset of recent works incorporates *post-hoc* explainability, most approaches either omit XAI or rely on limited visualization. In contrast, the proposed framework integrates comprehensive evaluation with multi-method explainability, providing both improved predictive performance and more reliable interpretability.

## 7 Confidence interval analysis

To assess the statistical reliability of the reported performance, 95% confidence intervals (CIs) were estimated for the principal evaluation metrics using the independent test set comprising 804 gastrointestinal endoscopic images. The confidence interval for a metric (p) was computed by Equation 28

(28) $$CI_{95\%}=p\pm 1.96\sqrt{\frac{p\left(1-p\right)}{n}}$$

where *p* denotes the observed metric value, *n* represents the number of test samples, and 1.96 is the z-score corresponding to the 95% confidence level.

The resulting confidence intervals are summarized in **Table 8**. The proposed Hybrid ViT-L/32–MaxViT-L model achieved an accuracy of 96.38% (95% CI: 95.08–97.68%), precision of 96.02% (95% CI: 94.67–97.37%), recall of 95.74% (95% CI: 94.35–97.13%), F1-score of 95.88% (95% CI: 94.52–97.24%), specificity of 99.21% (95% CI: 98.60–99.82%), and AUC of 99.36% (95% CI: 98.82–99.90%). The relatively narrow confidence intervals indicate stable predictive performance and limited variability in the estimated metrics.

**Table 8:** Point estimates and 95% confidence intervals for the proposed hybrid model.

Metric	Value (%)	CI lower (%)	CI upper (%)
Accuracy	96.38	95.08	97.68
Precision	96.02	94.67	97.37
Recall	95.74	94.35	97.13
F1-score	95.88	94.52	97.24
Specificity	99.21	98.60	99.82
AUC	99.36	98.82	99.90

## 8 Statistical analysis

To determine whether the observed performance differences among the evaluated models were statistically significant, a nonparametric Friedman aligned-ranks test was performed across all evaluation metrics. For the gastrointestinal disease classification dataset, the Friedman aligned-ranks statistic was 75.64 with a corresponding p-value< 0.001, leading to rejection of the null hypothesis. This result confirms the presence of statistically significant performance differences among the competing architectures.

The ranking results obtained from the Friedman procedure are summarized in **Table 9**. The proposed hybrid transformer achieved the highest average rank score (81.00), followed by MaxViT-Large (74.67), Swin-UNETR (68.33), and Swin-T-Base (62.83). Recently introduced architectures such as FLATer (60.67) and TransMT-Net (49.50) also demonstrated competitive rankings, whereas DeiT-Base (4.83) and ViT-B/16 (8.17) occupied the lowest positions. These rankings indicate that the proposed model consistently maintained superior performance across the evaluated metrics rather than excelling on a single measure.

**Table 9:** Friedman aligned ranks-based ranking of evaluated models across performance metrics.

Rank	Rank score	Algorithm
1	81.00000	Proposed hybrid transformer
2	74.66667	MaxViT-Large
3	68.33333	Swin-UNETR
4	62.83333	Swin-T-Base
5	60.66667	FLATer
6	49.50000	TransMT-Net
7	47.50000	Pyramid ViT
8	36.66667	MedViT
9	33.33333	ReViT
10	27.83333	TransFuse
11	22.83333	ViT-L/32
12	16.83333	TransUNet
13	8.16667	ViT-B/16
14	4.83333	DeiT-Base

To further examine pairwise differences, Holm-adjusted *post-hoc* comparisons were conducted between the proposed model and each baseline architecture. The results are presented in **Table 10**. Statistically significant improvements (adjusted p< 0.05) were observed over DeiT-Base, ViT-B/16, TransUNet, ViT-L/32, TransFuse, ReViT, and MedViT. In contrast, no statistically significant differences were detected with MaxViT-Large, Swin-UNETR, Swin-T-Base, FLATer, TransMT-Net, or Pyramid ViT after multiple-comparison correction. These findings indicate that while several recent transformer-based architectures remain competitive, the proposed model achieved the strongest overall ranking and the most consistent performance across evaluation criteria.

**Table 10:** Holm-adjusted *post-hoc* pairwise comparisons between the proposed hybrid transformer and baseline models.

Comparison with proposed model	Statistic	Adjusted p-value	Result
DeiT-Base	5.40838	0.00000	H0 is rejected
ViT-B/16	5.17169	0.00000	H0 is rejected
TransUNet	4.55629	0.00006	H0 is rejected
ViT-L/32	4.13025	0.00036	H0 is rejected
TransFuse	3.77521	0.00144	H0 is rejected
ReViT	3.38467	0.00570	H0 is rejected
MedViT	3.14798	0.01151	H0 is rejected
Pyramid ViT	2.37874	0.10423	H0 is accepted
TransMT-Net	2.23673	0.12652	H0 is accepted
FLATer	1.44381	0.59517	H0 is accepted
Swin-T-Base	1.28996	0.59517	H0 is accepted
Swin-UNETR	0.89942	0.73685	H0 is accepted
MaxViT-Large	0.44971	0.73685	H0 is accepted

## 9 Ablation study

To quantify the contribution of individual design choices, a series of ablation and sensitivity analyses was conducted on the Kvasir 8-class dataset. The experiments examine the effects of backbone selection, fusion strategy, fusion embedding dimension, and fine-tuning strategy, followed by an assessment of model robustness under different train–validation–test partitioning schemes.

### 9.1 Backbone complementarity

Single-branch baselines show that MaxViT-L (A_2_) outperforms ViT-L/32 (A_1_), achieving 94.26% accuracy versus 92.73%. The component-wise results are reported in **Table 11**. This suggests that hierarchical multi-axis attention is particularly effective in capturing fine-grained mucosal structures and local texture variations relevant to gastrointestinal findings. However, combining both branches (A_3_–A_8_) consistently improves performance beyond either standalone backbone. Even simple average logit fusion (A_3_) increases accuracy to 94.88%, confirming that global token-level context from ViT and hierarchical spatial encoding from MaxViT provide complementary information. The improvement from A_1_/A_2_ to hybrid configurations validates the design hypothesis of representational complementarity.

**Table 11:** Component-wise ablation analysis of the proposed hybrid transformer on the Kvasir 8-class dataset.

ID	Variant/component change	Accuracy (%)	Precision (%)	Recall (%)	F1-score (%)	Specificity (%)	AUC
A1	ViT-L/32 only (single-branch)	92.73	92.11	91.86	91.98	97.89	0.9713
A2	MaxViT-L only (single-branch)	94.26	93.88	93.51	93.69	98.71	0.9810
A3	Hybrid (ViT + MaxViT) Avg logit fusion	94.88	94.32	94.01	94.16	98.82	0.9851
A4	Hybrid concat features + MLP (no gating)	95.41	94.92	94.58	94.75	98.96	0.9880
A5	Hybrid gated fusion (no projection; raw dims)	95.62	95.11	94.84	94.97	99.02	0.9894
A6	Hybrid projection to shared space + Avg fusion	95.09	94.55	94.22	94.38	98.90	0.9862
A7	Hybrid projection + Gated fusion (Proposed, no label smoothing)	96.05	95.71	95.38	95.54	99.12	0.9910
A8	Proposed full: Projection + gated fusion + dropout + label smoothing	96.38	96.02	95.74	95.88	99.21	0.9936

### 9.2 Impact of fusion strategy and shared feature projection

Using the ablation configurations reported in **Table 11**, the influence of different fusion strategies and feature projection mechanisms was further examined. Naïve average logit fusion (A_3_) provides only moderate improvement. Moving to feature concatenation followed by MLP (A_4_) further improves accuracy (95.41%) and F1-score (94.75%), indicating that deeper integration of representations enhances multi-class discrimination. Adaptive gated fusion (A_5_) yields additional gains (95.62% accuracy), demonstrating the importance of sample-dependent weighting between global and local representations. When projection to a shared latent space is incorporated (A_7_), performance improves further (96.05% accuracy; AUC = 0.991), confirming that alignment of feature distributions stabilizes fusion.

The full proposed configuration (A_8_), incorporating projection, gated fusion, dropout, and label smoothing, achieves the best overall performance (96.38% accuracy; F1 = 95.88%; AUC = 0.992). These results indicate that the largest gains arise from (i) dual-branch modeling and (ii) replacing static fusion with adaptive gating.

### 9.3 Effect of fusion embedding dimension

The sensitivity of the model to the fusion embedding dimension is summarized in **Table 12**. The impact of fusion embedding dimension *D_f_* was evaluated across 256, 512, 1024, and 2048. Performance improves as dimensionality increases from 256 to 1024, with peak accuracy (96.38%) achieved at *D_f_* = 1024. Increasing dimensionality further to 2048 does not yield meaningful improvement (96.31%), suggesting diminishing returns beyond moderate embedding sizes. Thus, *D_f_* = 1024 offers an optimal trade-off between representational capacity and parameter efficiency.

**Table 12:** Sensitivity analysis of shared fusion embedding dimension.

Df	Accuracy (%)	F1 (%)	AUC
256	95.41	94.78	0.9886
512	95.92	95.36	0.9901
1024 (used)	96.38	95.88	0.9936
2048	96.31	95.81	0.9923

### 9.4 Fine-tuning strategy

The performance obtained under different fine-tuning strategies is presented in **Table 13**. Three fine-tuning strategies were evaluated:

**Table 13:** Impact of fine-tuning strategy on model performance.

Strategy	Description	Accuracy (%)	F1 (%)
S1	Train head only (frozen backbones)	94.72	94.05
S2	Head warm-up + unfreeze last 25%	96.38	95.88
S3	Full fine-tuning from epoch 1	95.61	94.93

- S_1_: Train classification head only (frozen backbones)
- S_2_: Head warm-up followed by unfreezing the last 25% of layers
- S_3_: Full fine-tuning from epoch 1

Training only the head (S_1_) yields reduced performance (94.72% accuracy), indicating that domain adaptation of the backbone layers is necessary. Full fine-tuning from the beginning (S_3_) performs better (95.61%) but does not match the performance of staged fine-tuning (S_2_). The head warm-up strategy (S_2_) achieves the best performance (96.38%), suggesting that gradual unfreezing stabilizes optimization and prevents early catastrophic forgetting of pretrained representations.

### 9.5 Evaluation across different train–validation–test splits

To assess the robustness of the proposed model to dataset partitioning, the Hybrid ViT-L/32–MaxViT-L architecture was evaluated using multiple train–validation–test split configurations ranging from 50:10:40 to 80:10:10. The results are summarized in **Table 14**. A gradual improvement in performance was observed as the proportion of training data increased from 50% to 70%, indicating that the model benefited from additional training samples, enabling it to learn more discriminative feature representations. The highest performance was obtained with the 70:10:20 split, achieving 96.38% accuracy, 96.02% precision, 95.74% recall, 95.88% F1-score, 99.21% specificity, and 99.36% AUC. Although larger training partitions (75:10:15 and 80:10:10) were also examined, marginal reductions in performance were observed. This behavior may be attributed to the smaller independent test sets, which yield fewer stable estimates of generalization performance. Based on these results, the 70:10:20 configuration was selected for all subsequent experiments as it provided the most favorable balance between model learning and reliable performance evaluation.

**Table 14:** Effect of dataset splitting ratios on the performance of the proposed model.

Train (%)	Validation (%)	Test (%)	Training images	Validation images	Test images	Accuracy (%)	Precision (%)	Recall (%)	F1-score (%)	Specificity (%)	AUC (%)
50	10	40	2000	396	1604	93.84	93.27	93.51	93.09	98.89	98.14
55	10	35	2200	396	1404	94.42	94.08	93.86	93.97	99.01	98.46
60	10	30	2400	396	1204	95.12	94.78	94.56	94.67	99.08	98.78
65	10	25	2600	396	1004	95.74	95.31	95.08	95.19	99.15	99.02
**70**	**10**	**20**	**2800**	**396**	**804**	**96.38**	**96.02**	**95.74**	**95.88**	**99.21**	**99.36**
75	10	15	3000	396	604	96.12	95.76	95.51	95.63	99.18	99.24
80	10	10	3200	396	404	95.91	95.48	95.22	95.34	99.12	99.16

Bold values indicate the highest performance was obtained with the 70:10:20 split.

## 10 Computational complexity analysis

To evaluate the computational requirements of the proposed framework, the number of trainable parameters, FLOPs, GPU memory consumption, and inference speed were compared with those of competing transformer-based architectures (**Table 15**). Lightweight models such as Pyramid ViT and MedViT exhibited the lowest computational demands, whereas large-scale transformer architectures required substantially greater resources. Owing to the integration of both ViT-L/32 and MaxViT-L backbones together with adaptive gated fusion, the proposed model contains 518.6 million trainable parameters and requires 62.7 GFLOPs per inference. Consequently, it incurs the highest computational cost among the evaluated methods, with a GPU memory requirement of 12.4 GB and an inference speed of 58 FPS. Despite this increased complexity, the model achieved the best overall predictive performance, attaining 96.38% accuracy, 95.88% F1-score, 99.21% specificity, and 99.36% AUC. These results indicate that the additional computational cost is accompanied by consistent gains in classification performance, making the framework suitable for GPU-enabled gastrointestinal disease analysis and decision-support applications.

**Table 15:** Computational complexity and inference performance of the proposed model and competing transformer architectures.

Model	Parameters (M)	FLOPs (G)	GPU memory (GB)	FPS	Inference time (ms/image)
ViT-B/16	86.6	17.6	5.1	126	7.94
ViT-L/32	304.3	18.8	9.7	82	12.20
DeiT-Base	86.0	17.5	5.0	132	7.58
Swin Transformer-Base	87.8	15.4	5.4	138	7.25
TransUNet	105.2	22.8	6.8	102	9.80
MaxViT-Large	212.0	43.9	8.6	69	14.49
MedViT	63.7	12.2	4.3	164	6.10
TransFuse	98.4	21.1	6.4	109	9.17
Swin-UNETR	62.2	15.7	5.8	120	8.33
Pyramid ViT	61.4	10.7	4.5	176	5.68
FLATer	72.8	13.8	4.9	151	6.62
TransMT-Net	81.5	16.5	5.2	143	6.99
ReViT	89.3	18.1	5.5	134	7.46
Proposed hybrid transformer	518.6	62.7	12.4	58	17.24

## 11 Explainability analysis using multi-method XAI visualization

**Figure 8:**
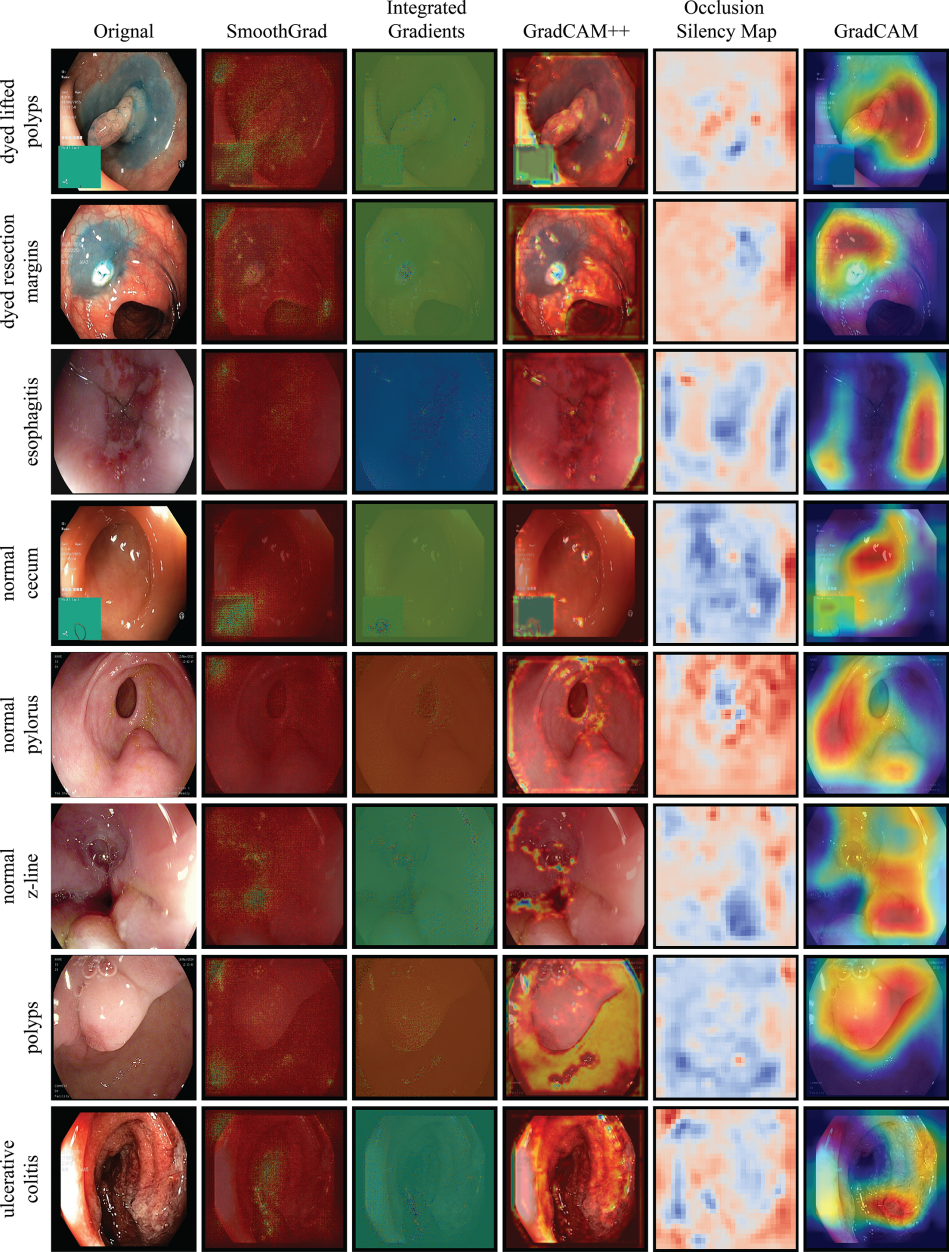
Multi-method explainability visualization of the proposed hybrid transformer across eight gastrointestinal disease classes.

**Figure 8** presents visual explanations for representative samples from eight gastrointestinal categories using five attribution methods: SmoothGrad, Integrated Gradients, GradCAM++, Occlusion Sensitivity, and GradCAM. Across lesion-centric classes—including dyed-lifted polyps, dyed resection margins, polyps, and ulcerative colitis—gradient-based localization methods (GradCAM and GradCAM++) consistently concentrate activation over visually salient pathological regions such as protrusions, dyed tissue boundaries, inflamed mucosa, and ulcerated surfaces.

In inflammatory classes such as esophagitis and ulcerative colitis, the highlighted regions correspond to erythematous or structurally disrupted mucosal areas rather than peripheral background tissue. For normal anatomical classes (normal cecum, normal pylorus, and normal z-line), activation maps predominantly localize around anatomical contours and luminal structures instead of diffuse or irrelevant areas.

Among the interpretability methods, GradCAM++ produces the most spatially focused and anatomically aligned heatmaps. GradCAM shows slightly broader activation patterns, while SmoothGrad produces more diffuse attribution distributions. Integrated Gradients generates low-contrast but spatially coherent maps. Occlusion sensitivity maps indicate influential regions but with comparatively coarse spatial resolution.

## 12 Discussion and clinical relevance

The experimental results indicate that the proposed hybrid architecture maintains stable and well-calibrated learning behavior across training. The close alignment between the training and validation curves, along with smooth loss convergence, suggests that integrating pretrained backbones with projection and gated fusion enables efficient optimization without overfitting. The consistent behavior of precision and recall further reflects balanced class discrimination, indicating that the model does not bias toward either sensitivity or specificity.

Analysis of the confusion matrix shows that misclassifications are limited and largely confined to visually similar or clinically related categories, rather than arbitrary errors. This pattern suggests that the model captures meaningful feature representations, although fine-grained differentiation among certain inflammatory conditions remains inherently challenging. Complementary ROC analysis demonstrates uniformly high AUC values, indicating strong separability across classes and stable performance across decision thresholds.

Class-wise evaluation confirms consistent performance across all categories, with high specificity and closely matched precision–recall values. The absence of class imbalance effects and the alignment between macro and per-class metrics indicate reliable generalization. Statistical testing further supports these observations, demonstrating that the performance improvements are not incidental but sustained across multiple evaluation measures, while remaining competitive with strong transformer baselines.

Comparative analysis highlights the advantage of combining global token-level reasoning with hierarchical spatial modeling. The adaptive gating mechanism contributes to these gains by regulating feature contributions based on input characteristics, rather than relying on fixed fusion. This is further supported by ablation results, where each component—particularly gated fusion and latent projection—provides measurable improvement.

The performance gains achieved by the proposed model are accompanied by increased computational complexity due to the simultaneous use of the ViT-L/32 and MaxViT-L backbones. Although this results in higher memory consumption and inference costs than lightweight transformer variants, the observed improvements in classification performance suggest that the additional representational capacity meaningfully contributes to disease discrimination. Such a trade-off may be acceptable in offline analysis or GPU-enabled clinical decision-support settings where diagnostic accuracy is prioritized over computational efficiency.

From a clinical perspective, the model demonstrates characteristics essential for deployment in gastrointestinal endoscopy. High recall supports reliable detection of pathological findings, reducing the risk of missed lesions, while high specificity limits unnecessary follow-up procedures. The consistency across classes enables multi-class stratification beyond simple detection, which is relevant for guiding diagnosis and intervention. Importantly, the interpretability analysis shows that model predictions are aligned with clinically meaningful regions, reinforcing confidence in its decision-making. Together, these findings suggest that the proposed framework offers a stable and clinically relevant foundation for computer-assisted gastrointestinal disease classification.

## 13 Limitations and further scope

Despite the promising performance of the proposed hybrid gated transformer framework, several limitations warrant acknowledgment.

First, the dual-branch architecture, which integrates ViT-L/32 and MaxViT-L, increases computational complexity, parameter count, and training time. Although this design enhances representational capacity, it may limit scalability in resource-constrained environments and restrict real-time deployment without model optimization or compression.

Second, the study was conducted using a single publicly available dataset. Although internal validation, evaluation under multiple train–validation–test partitioning schemes, ablation analysis, confidence interval estimation, and statistical significance testing were performed, the absence of external validation limits the generalizability of the findings. Variations in patient populations, imaging devices, acquisition protocols, and clinical settings may influence model performance. Consequently, the robustness of the proposed framework across diverse clinical environments remains to be established through validation on independent multi-center datasets and cross-dataset evaluation.

Third, the proposed framework operates on 2D still images, whereas gastrointestinal endoscopy is inherently dynamic. Frame-wise classification does not exploit temporal continuity or motion-related cues that may contribute to improved lesion characterization.

Fourth, although adaptive gated fusion provides sample-wise modulation of local and global representations, gating mechanisms introduce additional learnable parameters and may be sensitive to distribution shifts. The stability and calibration of gating under domain variation warrant further investigation.

Finally, preprocessing strategies such as fixed-resolution resizing and standard normalization may introduce scale distortion or color bias, potentially affecting lesion representation.

Future research should explore multimodal fusion incorporating clinical metadata, laboratory findings, or histopathology to enhance contextual reasoning. Extension toward video-based or 3D transformer architectures may improve spatiotemporal modeling. Model compression, pruning, or token-reduction strategies should be investigated to reduce computational overhead. Prospective clinical validation and real-time system integration remain critical steps toward practical deployment.

## 14 Conclusions

Accurate and reliable automated interpretation of gastrointestinal endoscopic images requires models that can reconcile fine-grained mucosal detail with broader anatomical and contextual cues. In this study, a dual-branch hybrid transformer architecture was developed to address this requirement by integrating global token-level modeling (ViT-L/32) with hierarchical spatial encoding (MaxViT-L) through an adaptive gated fusion mechanism. Rather than enforcing a fixed interaction between local and global representations, the proposed framework enables content-dependent modulation of feature contributions, reflecting the heterogeneous visual nature of gastrointestinal pathology. The experimental findings demonstrate that adaptive hybridization can enhance multi-class discrimination across inflammatory, polypoid, and normal anatomical categories without compromising training stability. Detailed class-wise evaluation and statistical comparison indicate that the performance gains are attributable to structured fusion rather than model scale alone. The ablation analysis further clarifies the incremental value of gated integration over single-stream or rigidly fused alternatives. Explainability was examined using multiple complementary *post-hoc* XAI approaches to assess whether model attention aligned with clinically plausible regions. The visual explanations consistently localized relevant mucosal patterns, supporting interpretability and reinforcing confidence in the model’s decision rationale. Although these explanations remain *post-hoc* and not causally grounded, their stability across classes strengthens the case for clinical interpretability. The present work advances transformer-based gastrointestinal image classification by introducing a controlled, sample-adaptive fusion strategy supported by rigorous validation. Continued evaluation across multi-institutional datasets and integration into real-world endoscopic workflows will be necessary to determine its practical clinical impact.

## Data Availability

The original contributions presented in the study are included in the article/supplementary material, further inquiries can be directed to the corresponding author/s. The code used in this study is uploaded to a GitHub repository. It can be accessed publicly at https://github.com/Shahid92-Phd/Gastrointestinal-Disease-Detection.git.
